# Transcriptome and organellar sequencing highlights the complex origin and diversification of allotetraploid *Brassica napus*

**DOI:** 10.1038/s41467-019-10757-1

**Published:** 2019-06-28

**Authors:** Hong An, Xinshuai Qi, Michelle L. Gaynor, Yue Hao, Sarah C. Gebken, Makenzie E. Mabry, Alex C. McAlvay, Graham R. Teakle, Gavin C. Conant, Michael S. Barker, Tingdong Fu, Bin Yi, J. Chris Pires

**Affiliations:** 1 Division of Biological Sciences, University of Missouri, Columbia, MO 65211 USA; 2 National Key Lab of Crop Genetic Improvement, Huazhong Agricultural University, 430070 Wuhan, Hubei P. R. China; 3 Department of Ecology & Evolutionary Biology, University of Arizona, Tucson, AZ 85721 USA; 4 Department of Biology, University of Central Florida, Orlando, FL 32816 USA; 5 Bioinformatics Research Center, North Carolina State University, Raleigh, NC 27695 USA; 6 Department of Ecology and Evolutionary Biology, Cornell University, Ithaca, NY 14850 USA; 7 School of Life Sciences, University of Warwick, Coventry, CV4 7AL UK

**Keywords:** Genome evolution, Plant evolution, Agricultural genetics

## Abstract

*Brassica napus*, an allotetraploid crop, is hypothesized to be a hybrid from unknown varieties of *Brassica rapa* and *Brassica oleracea*. Despite the economic importance of *B. napus*, much is unresolved regarding its phylogenomic relationships, genetic structure, and diversification. Here we conduct a comprehensive study among diverse accessions from 183 *B. napus* (including rapeseed, rutabaga, and Siberian kale), 112 *B. rapa*, and 62 *B. oleracea* and its wild relatives. Using RNA-seq of *B. napus* accessions, we define the genetic diversity and sub-genome variance of six genetic clusters. Nuclear and organellar phylogenies for *B. napus* and its progenitors reveal varying patterns of inheritance and post-formation introgression. We discern regions with signatures of selective sweeps and detect 8,187 differentially expressed genes with implications for *B. napus* diversification. This study highlights the complex origin and evolution of *B. napus* providing insights that can further facilitate *B. napus* breeding and germplasm preservation.

## Abstract (web summary)

Despite the economic importance of the allotetraploid crop *Brassica napus*, our knowledge of its phylogenomic relationships, genetic structure, and diversification is limited. Here, the authors show its complex origin and diversification by analyzing transcriptome and organellar sequencing data.

## Introduction

B*rassica napus* is an allopolyploid species (AACC, 2*n* = 38) thought to have formed by hybridization of two diploid species, *Brassica rapa* (AA = 20) and *Brassica oleracea* (CC = 18), between 6800 and 12,500 years ago^1–3^. It is a versatile crop with global economic importance. There are three currently recognized subspecies: *B. napus* subsp*. oleifera* (rapeseed or rape), *B. napus* subsp. *rapifera* (rutabaga or swede), and *B. napus* subsp. *pabularia* (Siberian kale or leaf rape)^1,4^. Rapeseed is a major vegetable oil resource, ranking second in worldwide oilseed production^5^. Cultivation of rapeseed started in Europe in the thirteenth century and became an important source of high-quality lubricant oil during the industrial revolution^6^. In the past half-century, double-low (low erucic acid and low glucosinolate) rapeseed was produced by intensive breeding^6,7^. Today, rapeseed is also important for edible oil production and livestock forage. Rutabaga also has a long history, with the first printed record traced back to 1620^8^. Mainly cultivated in Europe and North America, rutabaga can be used both as a root vegetable and as fodder. The leafy vegetable morphotype Siberian kale is cultivated worldwide for human consumption^9^. Collectively, these crops are valued at more than 41 billion U.S. dollars for annual production^5^.

Although the progenitors of *B. napus* are thought to be *B. rapa* and *B. oleracea*, the specific morphotypes that initially hybridized has yet to be identified. Currently, *B. napus* is believed to originate from the Mediterranean basin or some agricultural regions such as northern or western Europe, where *B. rapa* and *B. oleracea* co-exist^10,11^. Some molecular data, like RFLP and AFLP markers, indicate that *B. napus* was formed by several independent hybridization events^12–14^. However, whether the progenitors of *B. napus* were domesticated or wild is still unknown. According to several analyses of chloroplast and mitochondrial genomes, *Brassica montana* or other unknown Brassica species (2*n* = 18) may also have contributed to the *B. napus* C genome as a maternal donor^12,13,15^. These Brassica species are also referred to as *B. oleracea* wild relatives or wild C species as they have the same genomic structure as *B. oleracea*. However, there are also studies based on Brassica chloroplast genomes that have found that *B. rapa* is the maternal parent of *B. napus*, and furthermore, that *B. rapa* ssp. *broccoletto* (or ‘Spring Broccoli Raab’) is most closely related to the A genome ancestor of *B. napus*^15,16^. Many introgressions have been found both from *B. rapa* to *B. napus* and *B. napus* to *B. rapa*^12,17^, which further complicate the interpretations of these data.

Even though *B. napus* has a relatively short domestication history compared to *B. rapa* and some cereal crops like maize and Asian rice^9,18^, it has been domesticated into three distinct morphotypes: rapeseed, rutabaga, and Siberian kale. These types can be further clustered based on their growth habits into the winter types that need vernalization and the spring types that do not. *B. napus* can be also divided according to geographic region into European, North American, Asian, and Australian accessions. To date, almost all the studies on the diversity of *B. napus* crops have focused on the rapeseed subspecies. Of these studies, some find that the growth habit of *B. napus* better reflects its genetic structure than geographic origin^7,19–21^, whereas other studies suggest that geographic origin is more significant^13,22^. Furthermore, previous research has yielded differing opinions on the genetic diversity of *B. napus*, including an ongoing debate over which type of *B. napus* (winter or spring, rapeseed, or rutabaga) exhibits higher nucleotide diversity^7,20,23,24^. However, one consistent observation is that the A and C sub-genomes of *B. napus* have different degrees of genetic diversity^7,20^.

Understanding the origin and diversification of *B. napus* is important for germplasm preservation and for breeders to develop heterotic groups^25^. Therefore, we perform a comprehensive study of 183 accessions of *B. napus* and 174 accessions of potential progenitors. These include representatives of the major morphotypes across a global geographic distribution to determine the origin of *B. napus* by analyzing genomic SNPs and performing de novo assemblies of chloroplast and mitochondrial genes. We investigate *B. napus* selective sweep regions, and discuss differentially expressed genes (DEGs) highly related to the diversification process of *B. napus*. We further identify candidate genes under selection and unique DEGs for functional analysis associated with root tuber formation, leaf shape, and vernalization.

## Results

### RNA-seq and SNP identification

Our dataset includes 183 *B. napus* accessions, 112 *B. rapa* accessions, 42 *B. oleracea* accessions, 20 *B. oleracea* wild relatives (*B. hilarionis*, *B. villosa*, *B. montana*, *B. macrocarpa*, *B. rupestris*, *B. incana*, *B. insularis*), and five other Brassicaceae species as outgroups (Supplementary Data 1). Collectively these represent the main phenotypic diversity in each species. For *B. napus*, this panel represents all the major production countries and phenotypes: spring rapeseed, winter rapeseed, semi-winter rapeseed, rutabaga, and Siberian kale. Quality-filtered reads were mapped to the *B. napus* Darmor-*bzh* reference genome (version 4.1)^1^ with an average overall mapping rate of 77.28% in *B. napus*, 45.73% in *B. rapa*, 63.91% in *B. oleracea* and its wild relatives, and 22.80% in outgroups. This indicates that the C genome is more conserved than the A genome in *B. napus*, consistent with previous studies^7,20^.

We obtained 372,546 high-quality SNPs across all *B. napus* accessions for diversity and selection analyses. 95.03% (354,033) of these SNPs were located in genes, which matches our expectation for RNA-Seq data. The A sub-genome contained 219,164 (58.83%) SNPs and the C sub-genome comprised the remaining 153,382 (41.17%) SNPs. The C sub-genome is larger than the A sub-genome; however, more SNPs are found in the A sub-genome due to its higher nuclear diversity. The SNP density varied from 0.286 per kb on chromosome C02 to 1.080 per kb on chromosome A03. The most and fewest SNPs were found on A09 (29,627) and C02 (13,240), respectively (Supplementary Table 1). After further filtering, 36,829 synonymous and non-coding SNPs for *B. napus* were identified and used for genetic structure analysis. Then we added outgroups to all *B. napus* accessions and filtered the SNPs with the same pipeline to do the phylogenetic analysis. Similarly, 24,193 and 23,387 synonymous and non-coding SNPs were used to construct the A sub-genome and C sub-genome phylogenies, respectively.

We also used the rapeseed pan-transcriptome^26^ as a reference to recover 47,553 synonymous and non-coding SNPs across all *B. napus* accessions. As with the Darmor-*bzh* genome, the A genome (25,569 SNPs) showed more SNPs than the C genome (21,984 SNPs). To assess the role of the reference in determining the genetic clusters, these SNPs were also used to test for *B. napus* genetic structure and phylogenetic relationships.

### Genetic structure and phylogenetic relationship

**Fig. 1:**
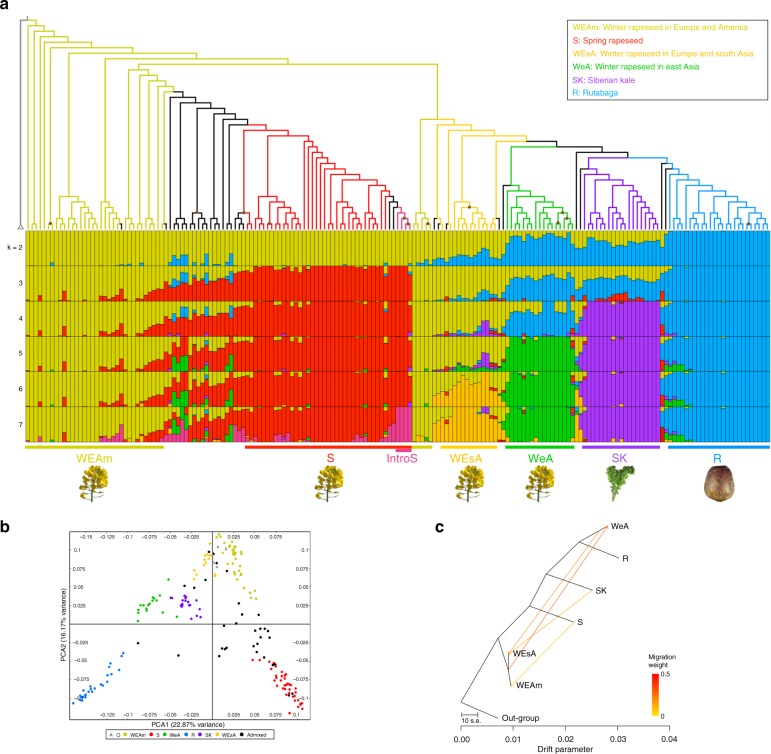
Population genetics analyses of 183 *B. napus* accessions. **a** Genetic structure and maximum-likelihood phylogeny of *B. napus*. Five other species from Brassicaceae used to root the phylogenetic tree are shown as a single branch. The fastSTRUCTURE ancestry kinship (*K*) is shown from 2 to 7. Colors indicate different genetic clusters. Yellow: WEAm; Red: S; Orange: WEsA; Green: WeA; Purple: SK; Blue: R; Pink: IntroS (Introgressed spring rapeseed); Black: genetically diverse accessions; Dark grey: outgroup. Brown dot: bootstrap support values <70%. **b** Principal component analysis of the 183 *B. napus* and five outgroup accessions. The *B. napus* color chart is the same as in **a**. Grey triangles were added to represent outgroups. **c** Treemix analysis of the six *B. napus* genetic clusters. Arrows represent the direction of migrations. Source data of Fig. 1a are provided as a Source Data file

FastSTRUCTURE^27^ was used to investigate the genetic structure of *B. napus* for clusters (*K*) from 1 to 10 based on the two reference sequences. When applied to the SNPs based on Darmor-*bzh* genome (36,829 SNPs), we found that the marginal likelihood plateaued at *K* = 5, and the cluster best fit for this data was identified as *K* *=* 7 (Supplementary Fig. 1). At the plateau of marginal likelihood, we identified five genetic clusters, including Winter rapeseed in Europe and America (WEAm), Rutabaga (R), Spring rapeseed (S), Siberian kale (SK), and Winter rapeseed in East Asia (WeA, or semi-winter rapeseed) (Fig. 1a, Supplementary Fig. 2, Supplementary Data 2). After that, the marginal likelihood value slightly increased at *K* = 6 and 7, and reached the peak at *K* = 7. When *K* = 6, Winter type rapeseed in Europe and South Asia (WEsA) were observed to cluster together. We found that at *K* = 7 that four spring type accessions (marked as IntroS) appeared to share genetic structure with other spring rapeseed; however, they did not differ from the other spring type accessions in phenotype or geographic location and were therefore kept in the S cluster. In addition to these six main clusters, some genetically diverse accessions (the main genetic component was less than 0.6 in fastSTRUCTURE) were also identified in our analyses; some of these accessions were recently admixed or resynthesized during the breeding process.

The genetic structure based on using the rapeseed pan-transcriptome as a reference for mapping (47,553 SNPs) also showed that the marginal likelihood plateaued at *K* = 5, and identified the same five genetic clusters as described above (Supplementary Fig. 3, Supplementary Fig. 4). Interestingly, the IntroS, observed at *K* *=* 7 with the Darmor-*bzh* genome (Fig. 1a), is observed at *K* = 6 in the analysis based on the rapeseed pan-transcriptome (Supplementary Fig. 4). This IntroS may be the result of introgression as indicated by the chloroplast data described below. Thus, using either the Darmor-*bzh* or pan-transcriptome as a reference, we recover the same six genetic clusters with increasing *K* (Fig. 1a, Supplementary Fig. 4). Given this agreement in the major clustering from both analyses, we suggest the recognition of six genetic clusters in *B. napus* as: WEAm, R, S, SK, WeA, and WEsA.

Using five closely related species (*Brassica nigra*, *Crambe hispanica*, *Sinapis alba*, *Eruca sativa*, and *Sisymbrium irio*) as outgroups, a maximum-likelihood phylogeny was constructed to resolve the relationships among the different *B. napus* subspecies (Fig. 1a). The phylogeny is highly congruent with our genetic structure results of six clusters, with two paraphyletic groups (WEAm and WEsA) and four monophyletic groups (R, S, SK, and WeA). Using the Darmor-*bzh* genome, we recover the WEAm cluster as sister to all other *B. napus* subspecies. The other five genetic clusters (S, WEsA, WeA, SK, and R), identified in the fastSTRUCTURE analysis, form two clades, each containing some WEAm accessions and other genetically diverse accessions. One of these grades contains all the S accessions and the other is composed of R, SK, WeA, and WEsA genetic clusters. A second phylogeny using the same outgroups, but with the resulting SNPs from mapping to the rapeseed pan-transcriptome^26^ was constructed. It showed the same two paraphyletic groups (WEAm and WEsA) and the same four monophyletic groups (R, S, SK, and WeA), as well as all S accessions in a clade sister to all other winter type *B. napus* (WEAm, R, SK, WeA, and WEsA; Supplementary Fig. 5). However, we also noticed some inconsistent patterns between these two phylogenies (Fig. 1a, Supplementary Fig. 5), including the relationships among R, SK, and WeA, and the placement of WEAm accessions.

In summary, using both references, we show the same six genetic clusters: two paraphyletic groups (WEAm and WEsA) and four monophyletic groups (R, S, SK, and WeA). Given the similarity of these results, we feel confident in using six groups for the remaining analyses below.

The genetic structure and phylogenetic relationships were also congruent with results from the principal component analysis (PCA) (Fig. 1b). In the PCA plot, all accessions assigned to clusters for *B. napus* were found to cluster into the six previously identified groups. These lineages’ migration histories was examined among the six genetic clusters using Treemix^28^, which implements composite likelihood to identify the most likely population splits. Individuals that phylogenetically clustered and had a consistent phenotype with these six genetic clusters were used in these analyses. Without migration (m = 0), the Treemix result explained 85.51% of the variance in SNP data. When we added migration events for four migration edges (m = 4) we were able to account for 99.74% of the variance (Fig. 1c). This graph model inferred four gene flows: from S to WEAm, from SK to WEsA, from WeA to WEsA, and from WEAm to WeA (Fig. 1c). This observation may explain the genetically diverse accessions in the genetic structure results.

Overall, the genetic structure and PCA of *B. napus* was found to correspond more to morphotype and growth habits than geographic origin, which is similar to other studies^7,19,20^. Consistent with insights from previous studies of *B. napus* origin^1,3,18^, our phylogeny based on Darmor-*bzh* genome also indicates that the WEAm genetic cluster was most likely the first formed morphological subgroup. The order of the genetic clusters fall out in the fastSTRUCTURE analysis is consistent with written records: the first references of *B. napus* are winter rapeseed (1578), rutabaga (1620), and spring rapeseed (~1700)^8,11^. However, while our inferred phylogeny and those from previous studies generally agree on the genetic clusters, they are incongruent with each other with respect to the relationships among these clusters^4,12,13,16^. This divergence may be caused by the different germplasm sets used, the variety of methods employed, or different reference sequences used in these studies. The inconsistent relationship of rutabaga (R), Siberian kale (SK), and semi-winter rapeseed (WeA) indicates that a *B. napus* pan-genome that can represent all the morphotypes is greatly needed. With these genomes, we can access homoeologous genome exchanges among different subspecies easier. Admixture was widespread among different *B. napus* clusters, especially in rapeseed subspecies, which may be due to efforts by breeders or the result of different genetic clusters being grown in close geographic proximity. Breeder-associated admixture between different genetic clusters has often been used to create heterosis and disease resistance; for example, crossing spring or European winter lines with Chinese semi-winter lines^29,30^.

### Relationships among *Brassica napus* and its progenitors

**Fig. 2:**
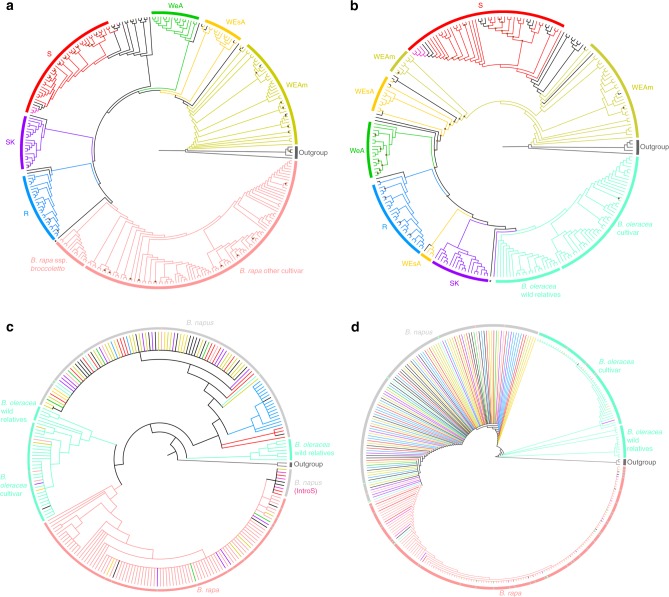
Phylogenetic analyses of *B. napus* and its progenitors. Maximum-likelihood phylogenetic tree constructed among: **a** *B. napus* and *B. rapa* based on the A sub-genome (24,193 SNPs); **b** *B. napus*, *B. oleracea*, and wild C species based on the C sub-genome (23,387 SNPs); **c** Chloroplast phylogeny of *B. napus* and all its progenitor species (*B. rapa*, *B. oleracea*, and wild C species) using 62 chloroplast genes; **d** Mitochondria phylogeny of *B. napus* and all its progenitor species using 42 mitochondrial genes. *: resynthesized *B. napus* accession New Hakuran; WEAm: Winter rapeseed in Europe and America; S spring rapeseed, WEsA winter rapeseed in Europe and South Asia, WeA winter rapeseed in East Asia, SK Siberian kale, R rutabaga, IntroS Introgressed spring rapeseed. Brown dot: bootstrap support values <70%; **c**, **d** were made with a bootstrap cut off value of 70%

We generated two maximum-likelihood (ML) phylogenies based on the A and C sub-genome separately. Based on the A sub-genome phylogeny, we studied the relationship between *B. napus* and 112 accessions of *B. rapa*. The phylogeny again showed six genetic clusters among *B. napus* samples and together are sister to a monophyletic *B. rapa*. A newly resynthesized *B. napus* accession New Hakuran was found to cluster with *B. rapa*, a pattern, which is known to occur with newly formed accessions^13^ (Fig. 2a, Supplementary Fig. 6). Based on the C sub-genome, we investigated the relationship between *B. napus* and 62 accessions of *B. oleracea* as well as the wild relative species. Although the wild C species and *B. oleracea* cultivars were clearly separated into two branches, they were recovered as a monophyletic group sister to a *B. napus* clade excluding the newly resynthesized New Hakuran and a Siberian kale accession (Fig. 2b, Supplementary Fig. 7). When compared with the A and C sub-genome phylogenies constructed based on the rapeseed pan-transcriptome, incongruences were found (Supplementary Fig. 8). Since 88.22% (102,422 out of 116,098) CDS models of the rapeseed pan-transcriptome are from *B. rapa* and *B. oleracea* genomes^26^, it indicates that the components of the reference sequences have non-negligible impact on the topology of phylogenies. However, although the relationships among these clusters differed, all analyses again consistently recovered six genetic clusters in both the A and C sub-genome phylogenies with the both reference sequences.

We also explored the relationships between *B. napus* and its progenitors based on each chromosome. These 19 phylogenies mainly showed the same species level relationships as the sub-genome except three C sub-genome chromosomes C01, C02, C05 (Supplementary Fig. 9). This may be the result of recent sub-genome rearrangement, as C01 and C02 have the most frequent homoeologous exchanges^31^. Another possibility for this phenomenon could be interspecies introgression, as seen from *Raphanus raphanistrum* to *B. napus*^32^.

Subsequently, the chloroplast and mitochondrial genes of *B. napus* and progenitors were de novo assembled using data generated by genome survey sequencing (GSS). We constructed a ML phylogeny with both chloroplast and mitochondrial data. In the chloroplast phylogeny, two wild C species, *B. villosa* and *B. rupestris*, were found to be sister to all other species. Additionally, the *B. napus* clade was separate from *B. oleracea*, wild C, and *B. rapa* (Fig. 2c, Supplementary Fig. 10). Unlike the nuclear genome, we were generally unable to distinguish subspecies based on the chloroplast phylogeny: the only subspecies that was monophyletic was rutabaga (R). In addition, some *B. napus* accessions clustered in the *B. rapa* clade or *B. oleracea* clade and vice versa. It is interesting to note that IntroS was classified in the *B. rapa* clade (bootstrap support > 70%; Fig. 2c, Supplementary Fig. 10). This association suggests a recent introgression between *B. rapa* and *B. napus*, since spring rapeseed is a recently formed subgroup. This introgression event makes this small spring rapeseed group different from other spring rapeseed in both its nuclear and organellar genome. The mitochondrial phylogeny, similar to the chloroplast phylogeny, showed the wild C species as a single clade. The remaining samples clearly divided into three clades: *B. oleracea*, *B. napus*, and *B. rapa* (Fig. 2d, Supplementary Fig. 11). Some mixed clusters among the three species were also observed.

The proposed multiple origins of *B. napus* is not confirmed based on the nuclear phylogenies, which is consistent with previous studies using nuclear markers^13,15^. Similar to Song et al. (1992)^13^, we found only newly resynthesized *B. napus* clustered directly with its progenitors. Our results suggest that perhaps we have not sampled the extant populations of *B. rapa* and *B. oleracea* that are most similar to the ancient progenitors of *B. napus*, or that the progenitor populations may be extinct^33^. Alternatively, it is also possible that after several thousand years of evolution, the *B. napus* nuclear genome has diverged beyond easy recognition from its progenitors’ nuclear genomes. The topological difference of these six genetic clusters in A and C sub-genomes also indicates that there was asymmetric sub-genome evolution during their diversification. Compared to the nuclear phylogenies, the organellar phylogenies of *B. napus* are too conserved to distinguish these six genetic clusters. Interestingly, our chloroplast result matches the nuclear genome-based Treemix^28^ result, which showed no significant admixture between rutabaga (R) and other genetic clusters. Our chloroplast results differ from previous studies that found a multi-origin of *B. napus* using haplotypes of the chloroplast genome^13,15^. It is common for breeders to cross *B. rapa* with *B. napus* to make *B. napus* more fit in local environments or have better agronomic traits^34,35^, which may contribute to the introgression identified in the chloroplast phylogeny. Apart from known crosses, it is difficult to differentiate mixed clusters found among *B. napus* and its progenitors are due to natural hybridizations or breeder mediated introgression.

### Nucleotide and gene expression changes via diversification

**Fig. 3:**
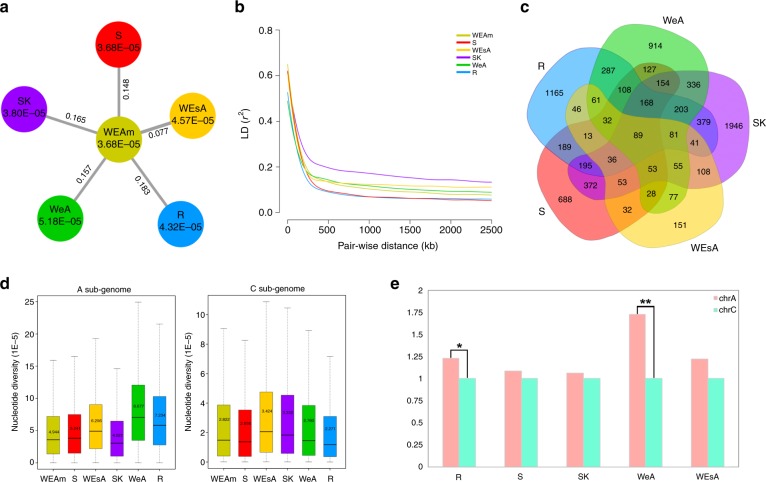
Comparison of different *B. napus* genetic clusters. **a** Nucleotide diversity and F_ST_ across six genetic clusters. The value in each circle shows nucleotide diversity for this cluster; the value on each line represents the F_ST_ between WEAm and another cluster. **b** Decay of linkage disequilibrium (LD) in each genetic cluster measured by *r*^*2*^. **c** Venn diagram of differentially expressed genes (DEGs) between WEAm and other genetic clusters. **d** Nucleotide diversity in each genetic cluster based on separated A and C sub-genomes. The box represents the interquartile range. Band inside each box represents median. Number inside each box represents the mean value. The whiskers represent the minimum and maximum values. **e** The ratio of (DEGs in A sub-genome)/(DEGs in C sub-genome). *p-value < 0.05 (*χ*^2^ test); **p-value < 0.01 (*χ*^2^ test). 372,546 SNPs were used for all the analysis in **a**, **b**, **d**. Source data of Fig. 3e are provided as a Source Data file

We examined population divergence among the six different genetic clusters by calculating their fixation statistics (F_ST_). The results indicate Rutabaga (R) had the largest population divergence from WEAm (0.183), while WEsA was found to have minimum divergence from WEAm (0.077) (Fig. 3a, Supplementary Table 2, Supplementary Fig. 12). Based on nucleotide diversity (π) for each genetic cluster, we found WEAm and spring rapeseed (S) had the lowest and equal nucleotide diversity (π = 3.68 × 10^−5^), closely followed by Siberian Kale (SK) (π = 3.80 × 10^−5^), while WeA showed the highest nucleotide diversity (π = 5.18 × 10^−5^) (Fig. 3a). Linkage disequilibrium (LD) analyses revealed that SK had the largest LD value, followed by WEAm, WEsA, and WeA (the three winter rapeseed clusters); R and S had the lowest LD value (Fig. 3b).

The gene expression analysis in leaf tissue between WEAm and five other genetic clusters showed that 8,187 genes were differentially expressed genes (DEGs). SK had the most DEGs (4,269) with R (3,093) also showing a high number of DEGs (Supplementary Data 3, Supplementary Table 3). WeA and S had similar DEGs with 2,773 and 2,337 genes separately. Meanwhile, WEsA, whose phenotype was more similar with WEAm, only had 956 DEGs. Since R and SK clusters had observably different morphotypes with WEAm, S and WeA had distinguishable growth habits with WEAm, these results were reasonable. Interestingly, there were significantly more down-regulated DEGs than up-regulated DEGs (*t*-test, *p* < 0.05) (Supplementary Table 3). We found 4,864 DEGs were unique to different genetic clusters. Following a similar pattern to the total DEGs in each cluster, SK (1,946) and R (1,165) had more unique DEGs than WeA (914) and S (688), and WEsA (151) had fewer unique DEGs than the others (Fig. 3c). When using the rapeseed pan-transcriptome, the same patterns as described above were identified, suggesting that while differences in the reference sequence have profound effects on phylogeny, it does not at population level analyses. (Supplementary Fig. 13). The number of DEGs varied among chromosomes as well. Generally, A03, A09, C03 had more DEGs than the others and A04, A08 had fewer DEGs (Supplementary Fig. 14).

Nucleotide diversity and DEGs were analyzed based on A and C sub-genomes as well. Generally, the A sub-genome had higher genetic diversity than the C sub-genome (Fig. 3d), again agreeing with previous studies^7,20^. Based on the A sub-genome, WeA had the most genetic diversity while SK had the least (Supplementary Table 4). However, based on the C sub-genome, WEsA and R showed the highest and lowest nucleotide diversity, respectively. Besides, the A sub-genome obtained more DEGs than the C sub-genome, especially in WeA and R (*χ*^2^ test, *p* < 0.01 and *p* < 0.05, respectively) (Fig. 3e). The genetic diversity and DEGs difference in the A and C sub-genome among each genetic cluster also indicated asymmetric sub-genome evolution during their diversification. This phenomenon is also reported in paleopolyploid *B. oleracea* and allotetraploid cotton^36,37^. The unique DEGs obtained in each genetic cluster may play significant roles in their differences in growth habit or morphotype.

Previous studies have found inconsistent rankings of genetic diversity among different subgroups^7,20,23,24^. Bus et al. (2011) found that the recently released winter rapeseed inbred lines have lower genetic diversity than lines of winter rapeseed that have been inbred and released several decades ago^24^. In our study, both Winter rapeseed in Europe and America (WEAm) and spring rapeseed (S) have the lowest genetic diversity. This low diversity may have been caused by intensive breeding for better seed quality, like double-low rapeseed, followed by huge population expansion in Europe and Canada^38,39^. The selective pressures experienced by spring rapeseed (S) may also explain the faster LD decay in S compared to winter rapeseed, a hypothesis consistent with Delourme et al. (2013)^23^. The other genetic clusters with higher genetic diversity may be due to the recent introgression by breeders. For example, when breeders introduced *B. napus* to East Asia, they performed crosses of *B. rapa* to *B. napus* to generate *B. napus* cultivars that were better adapted to the local environment^40^. These breeding practices most likely contributed to WeA having the most genetic diversity in the A sub-genome.

### Identification of selective sweep regions

**Fig. 4:**
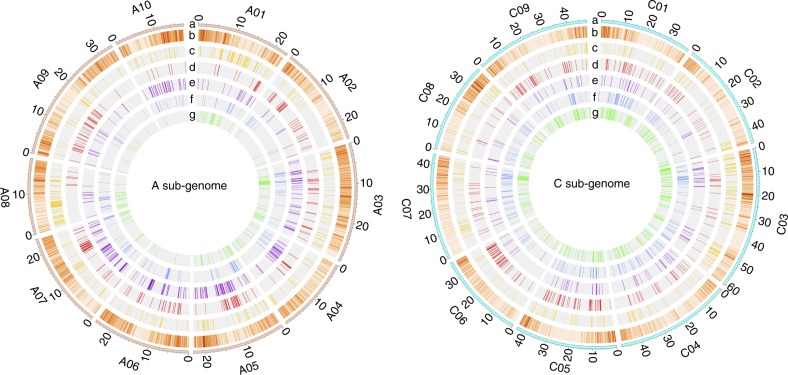
Selective sweep regions among different genetic clusters. Left panel is A sub-genome and right panel is C sub-genome, each with rings labeled **a**–**g**. **a** A and C chromosomes of *B. napus*. **b** SNP density within a 200 kb window across each chromosome. **c–g** selective sweep regions. Colors indicate different genetic clusters. Red: S; Orange: WEsA; Green: WeA; Purple: SK; Blue: R

The diversification of *B. napus* resulted in changes in morphotype and growth habit, including variation in root expansion, leaf shape, and vernalization requirements. A total of 570, 474, 452, 420, and 412 selective sweep regions were both identified by XP-CLR and π ratio (π_WEAm_/π_other_cluster_) methods in WEsA, S, WeA, R, and SK, which contained 3,391, 2,972, 2,619, 2,414, and 2,447 genes, respectively (Fig. 4, Supplementary Data 4). Although WEsA had the most selective sweep regions, the top 5% XP-CLR score (7) for WEsA is much lower than others (SK: 15, R: 14, S: 12, WeA:11) (Supplementary Fig. 15).

We were able to identify genes, which were important for the different morphotypes or growth habits in our selective sweeps. For example, we identified *BnaA06g18280D* and *BnaC03g38180D* whose homologs in its potential progenitor (*Bra038270*, *Bo3g065780*) functioned in tuber-forming in rutabaga (R)^41^. Additionally, 19 other candidate selected genes were identified that may function in forming tuberous roots in rutabaga, including three genes regulating auxin signaling, one gene functioning in auxin transport, and one gene contributing to auxin synthesis in roots (Supplementary Table 5, Supplementary Fig. 16). In leafy type Siberian kale (SK) we found *BnaA05g16600D*, which is homologous to candidate genes identified in *B. rapa* (*Bra033869*) and *Arabidopsis thaliana* (*AT1G31880*) that contribute to the curled leaf phenotype^41^. In addition, 10 selected genes were identified in SK, which are highly related to leaf shape and two genes functioning in cellular response to auxin stimulus (Supplementary Table 6, Supplementary Fig. 17). Additionally, five genes that function in flowering time and vernalization, *BnPHYA*, *BnEFS*, *BnSUF4*, *BnSPL3*, and *BnaA07g22720D*^7,42,43^, were found in selective sweep regions of spring rapeseed (S). Another 18 candidate genes that are believed to possibly regulate flowering time or vernalization in S were identified (Supplementary Table 7, Supplementary Fig. 18). Interestingly, among all these candidate genes we only identified one paralogous gene pair (*BnaA06g18130D* and *BnaC03g55810D*) in rutabaga (R), which are both under selective sweeps. Similar to the DEGs, many selective sweep regions were specific to a genetic cluster. Only 22 genes among the six selective sweep regions were found to be common among all genetic clusters (Supplementary Fig. 16, Supplementary Table 8). Even though most of these genes (21 of 22) are homologous with genes belonging to *B. rapa* and *B. oleracea*, more than half (12 of 22) of these genes were unable to be annotated with *A. thaliana* genes. This indicates that many of these important crop diversification related genes are Brassica specific.

More selective sweep regions were found in the C sub-genome than A sub-genome among all genetic clusters except SK (Supplementary Fig. 17). This implies that C sub-genome has undergone stronger selection than the A sub-genome, which is consistent with a previous study in rapeseed^40^. The numbers of selective sweeps also varied between different chromosomes across the genetic clusters. These results imply that A09, C05, and C06 may have had more selective pressure during the change of growth habit in *B. napus* (Supplementary Fig. 18).

Of the top 5% XP-CLR score, WEsA was found to have the lowest score compared to the other genetic clusters indicating most of the selective sweep regions in WEsA may play a role in crop improvement. In contrast, the other genetic clusters, with top 5% XP-CLR scores at least 11, contained more selective sweep regions contributing to the diversification of economically important organs such as the expanded root (rutabaga) or edible leaf (Siberian kale). Similar phenomena were also found in soybean^44^. *Brassica rapa*, *B. oleracea*, and *B. napus* all have root type (like turnip, kohlrabi, and rutabaga) and leafy type (like Chinese cabbage, kale, and Siberian kale) vegetables^11,18,41^. As in a previous study of *B. rapa* and *B. oleracea*^41^, we also found that the phytohormone, auxin, plays an important role in the new morphotype formation. Interestingly, the homologs of several of these genes in *B. rapa* and *B. oleracea* are also under positive selection during the domestication of tuber-forming and leaf curling^41^. These genetic clusters and genes can both be materials to study parallel evolution and be used in breeding processes^45^. Besides, these selective regions are good resources for specific agronomic trait introgression or admixture. CRISPR-Cas9 genome editing strategies may also be applied for these selected candidate genes for de novo diversifications of *B. napus*, similar to a study in tomato^46^. Our results showed A and C sub-genomes undergo different selective pressure, which indicates the asymmetric sub-genome evolution. Additionally, different chromosomes may have contributed unequally to different growth habits or morphotypes.

### Comparative genomic analyses of different crops

**Fig. 5:**
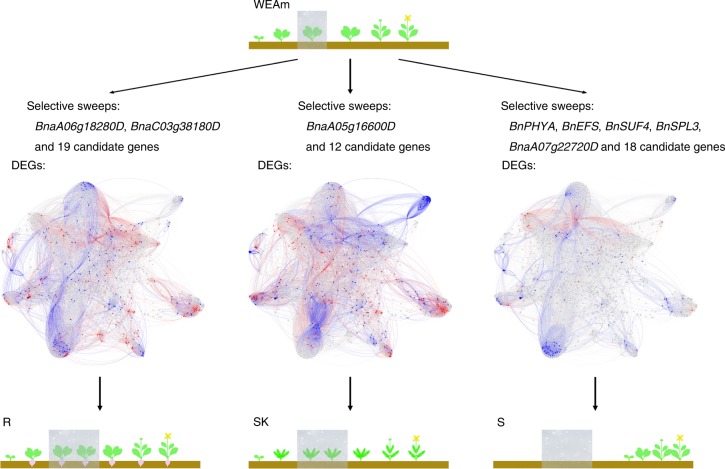
Different diversification processes of *B. napus*. Plant cartoons were modified from Schiessl et al. (2017)^43^ and show the growth cycle of different *B. napus*, the grey rectangle indicates winter (vernalization). The networks represent biochemical reactions that are catalyzed by enzymes (three middle panels); the colors of the nodes (reactions) in the metabolic networks represent the difference in expression of the *B. napus* genes that are orthologs to the *A. thaliana* genes encoding the enzymes for these reactions. Red: overexpressed comparing to WEAm; Blue: underexpressed comparing to WEAm; Grey: no unique DEGs detected. WEAm: Winter rapeseed in Europe and America, S: spring rapeseed, SK: Siberian kale, R: rutabaga

We further compared the functions of unique DEGs among the three most diversified genetic clusters, spring rapeseed (S), rutabaga (R), and Siberian kale (SK), using Gene Ontology categories and information about metabolic reactions. In addition to cell metabolism or housekeeping genes, we identified DEGs in these three genetic clusters commonly related to three phytohormones (abscisic acid, cytokinin, and jasmonic acid), temperature (response to cold), and chloroplast-related genes (Fig. 5, Supplementary Data 5, Supplementary Data 6). Additionally, there are some cluster-specific categories or reactions significantly enriched in different genetic clusters. For example, genes associated with the function of positive regulation of flower development were upregulated in spring rapeseed (S), which correlates to its early flowering phenotype. In Siberian kale (SK), we found upregulated genes that function in response to different light (red, blue, and far red) and some downregulated genes related to glucosinolate biosynthesis. We speculate that this difference may be responsible for the dark green and tasty leaf of Siberian kale. In addition, some upregulated DEGs in rutabaga (R) were identified as functioning in glucosinolate biosynthetic and catabolic processes.

These preliminary results indicate that phytohormones play an important role in the diversification of *B. napus* which is consistent with Cheng et al. (2016) study in leaf-heading and tuber-forming in *B. rapa* and *B. oleracea*^41^. Based on our phenotyping, spring rapeseed does not need vernalization, while rutabaga and Siberian kale need longer vernalization than winter rapeseed. Consequently, we found DEGs that function in response to cold in all three genetic clusters. Besides this global observation, the expression of some specific genes also changed due to different diversification processes, including the genes that regulate flower development in spring rapeseed and the genes that respond to different light in Siberian kale (SK). It is interesting that the glucosinolates and their products varied among different *B. napus* subspecies. This variation may be due to different artificial selection during their formation. For example, the expression of genes in the glucosinolate pathway was downregulated in Siberian kale, which may be due to consumers preferring leafy type vegetables without glucosinolates. Comparatively, rutabaga, a vegetable whose roots are consumed, may have high glucosinolate content in its leaf for better biotic resistance.

## Discussion

In our comprehensive study of *B. napus* morphotypes, we identified six genetic clusters of *B. napus* consistently across genetic structure, PCA, and phylogenetic analyses, regardless of reference sequence used for mapping. Spring rapeseed was found to be independently derived from winter rapeseed in Europe from the other four winter types of *B. napus*. We were unable to confirm the multi-origin hypothesis of *B. napus*; however, our comparisons of nuclear and organellar data suggest past introgressions between *B. napus* and its possible progenitors, as well as admixture among different *B. napus* genetic clusters. The population genetic analyses showed that these six genetic clusters have undergone different selective pressures, which match the known breeding histories. Using both nucleotide diversity and gene expression level, we show that *B. napus* has an asymmetric sub-genome evolution. We further identified candidate genes that went through selective sweeps underlying vernalization (spring rapeseed), leaf shape (Siberian kale), and root tuber-forming (rutabaga). The common and specific metabolic networks and GO functions we found among the main three morphotypes aids our understanding of *B. napus* diversification.

Our study can further facilitate *B. napus* breeding and germplasm preservation efforts. Candidate genes and pathways identified can be used for selecting agronomic traits. Genetic structure and phylogenetic results can assist breeders to find crossing groups to maximize heterosis. Finally, the high-quality SNPs can help breeders to conduct genome wide association studies and marker-assisted selection.

## Methods

### Sample collection and high-throughput sequencing

All the seeds used in this study were from U.S. Department of Agriculture (USDA), and the author’s personal collection (Supplementary Data 1). Seeds were planted in a Conviron growth chamber (16 h of light at 23 °C and 8 h of dark at 20 °C) with four replicates for each accession at the Bond Life Science Center at the University of Missouri (Columbia, MO, USA). The second youngest leaf of each plant was sampled and combined per accession for RNA and DNA isolation when the plants had five true leaves. After that, RNA was isolated using the PureLink^TM^ RNA Mini Kit (Invitrogen, USA), double-stranded cDNA was synthesized though Maxima H Minus Double-Stranded cDNA Synthesis Kit (Thermo, Lithuania) and then purified by GeneJET PCR Purification Kit (Thermo). A total of 277 RNA-Seq libraries were made using Illumina TruSeq kits and 2 × 250-bp paired-end sequencing was carried out on an Illumina HiSeq 2000 platform at the University of Missouri DNA core. Meanwhile, DNA, used for genome survey sequencing (GSS), was extracted using a Urea buffer method^47^ and 2 × 150-bp paired-end libraries were prepared by Illumina TruSeq kits and sequenced using the Illumina NextSeq system at the University of Missouri DNA core.

From each accession, leaf tissue was collected for genome size analyses and phenotype was recorded after flowering. By doing this, we identified 13 putatively *B. napus* accessions which were mislabeled and were actually *B. rapa*, *B. oleracea*, or *B. juncea*. Similarly, one accession identified as *B. oleracea* was really *B. rapa*, and three identified as wild C species (2 *Brassica cretica* and 1 *Brassica incana*) were actually *B. oleracea*. These cross-species mislabeled samples were discarded. Four *B. napus* were mislabeled with respect to their subspecies: these accessions were corrected in their identification and used in the study (Supplementary Table 9).

### Mapping and SNP calling

All raw paired-end RNA-Seq reads were filtered by Trimmomatic (version 0.36)^48^. *Brassica napus* reads were both mapped to the *B. napus* Darmor-*bzh* genome (version 4.1; http://www.genoscope.cns.fr/brassicanapus/) and rapeseed pan-transcriptome^26^, using TopHat2 (version 2.1.0)^49^ with Bowtie2 (version 2.2.6)^50^. To reduce the influence of mismapping between the A and C sub-genomes due to their high similarity, *B. rapa* reads were mapped to the A sub-genome of *B. napus* Darmor-*bzh* genome, while *B. oleracea* and other wild C species were mapped to the C sub-genome of *B. napus* Darmor-*bzh* genome using the pipeline described above. Only unique mapped reads were kept for further use. SNPs for each accession were called using HaplotypeCaller in GATK (version 3.7)^51^ as described by the GATK best practices workflow for SNP and indel calling using RNA-Seq data. Finally, we merged different accessions with GenotypeGVCFs in GATK.

We then constructed seven SNP groups for different analysis. Group I (372,546 SNPs): Based on Darmor-*bzh* genome reference. Combined all *B. napus* GVCFs and filtered with the following processes: SNPs with a mapping depth >10, a mapping quality >30 in vcffilter (https://github.com/vcflib/vcflib), and genotyping rate >50% and minor allele frequency (MAF) higher than 5% in PLINK (version 1.9)^52^. Group II (36,829 SNPs): Based on Darmor-*bzh* genome reference. Combined all *B. napus* GVCFs and filtered with the following processes: SNPs with a mapping depth >10, a mapping quality >30 in vcffilter (https://github.com/vcflib/vcflib), genotyping rate >90%, minor allele frequency (MAF) higher than 5% in PLINK (version 1.9)^52^, and only synonymous and non-coding SNPs as annotated by SnpEff (version 4.3)^53^. Group III (24,193 SNPs): Based on Darmor-*bzh* genome reference. Combined all *B. napus*, *B. rapa*, and outgroups GVCFs, SNPs called on A sub-genome and filtered by the same processes as Group II. Group IV (23,387 SNPs): Based on Darmor-*bzh* genome reference. Combined all *B. napus*, *B. oleracea*, wild C species, and outgroups GVCFs, SNPs called on C sub-genome and filtered by the same processes as Group II. Group V (47,553 SNPs): Based on rapeseed pan-transcriptome reference. Combined all *B. napus* GVCFs, SNPs then filtered by the same processes as Group II. Group VI (25,569 SNPs): Based on rapeseed pan-transcriptome reference. Combined all *B. napus*, *B. rapa*, and outgroups GVCFs, SNPs called on A genome and filtered by the same processes as Group II. Group VII (21,984 SNPs): Based on rapeseed pan-transcriptome reference. Combined all *B. napus*, *B. oleracea*, wild C species, and outgroups GVCFs, SNPs called on C sub-genome and filtered by the same processes as Group II.

To test for SNP ascertainment bias, we counted SNPs in each genetic cluster in each of these four groups based on Darmor-*bzh* genome. By using worldwide germplasm collections and the GATK best practices pipeline, the SNP numbers and the read-mapping rates of the WEAm genetic cluster, of which the *B. napus* reference genome belongs to, do not show significantly higher values when compare to the other five genetic clusters in all of the four SNP groups (Supplementary Table 10, Supplementary Fig. 19). This indicates that the SNP ascertainment bias, if present, is small and the SNPs we discovered are good for further downstream analyses^54^. However, our methodology based on RNA-Seq data does provide limited insight into structural rearrangement, which would require genomic structure data.

### Population structure and phylogenetic inference

To reduce influences of natural or artificial selection, SNPs only include synonymous and non-coding SNPs, were used for phylogenetic inference and population structure study. Since SNP Group II(36,829 SNPs) and Group V(47,553 SNPs) are obtained based on different reference sequences, they are used to test the reference bias of *B. napus*. The population structure among *B. napus* accessions was inferred by fastSTRUCTURE (version 1.0)^27^ based on a variational Bayesian framework. We tested *K* from 1 to 10 with five replicates in each *K* value using default convergence criterions and priors. After that, the best *K* value was estimated using chooseK.py in the fastSTRUCTURE package and all *K* values and their replicates were summarized by CLUMPAK^55^. Finally, the multiple *K* value results of fastSTRUCTURE were reorganized and visualized in Excel corresponding to the accessions order of phylogenetic inference. The maximum-likelihood phylogeny was constructed using RAxML (version 8.2.8)^56^ using the ASC_GTRGAMMA model with 100 bootstrap replicates. Bootstrap support values were then calculated by BOOSTER^57^. Five known closely related species (*Brassica nigra*, *Crambe hispanica*, *Sinapis alba*, *Eruca sativa*, and *Sisymbrium irio*) were used as outgroups.

PCA analyses were processed by PLINK (version 1.9) and visualized through Genesis (https://github.com/shaze/genesis). The same SNPs set was also used for Treemix (version 1.13)^28^ analyses. To reduce the bias by the recent admixture, only the accessions in these six genetic clusters with their main genetic component larger than 0.6, phylogenetically clustered together, and phenotype matched (Supplementary Data 2) were used in Treemix analyses. The same outgroups used in the phylogenetic analyses were applied here as well. One hundred SNPs were counted as resampling blocks to generate bootstrap replicates. Admixture trees were built with m = 1–10 migration events and the model fit for each migration event was evaluated by estimating the proportion of variance explained by each migration model among all the subgroups.

The maximum-likelihood phylogeny among *B. napus* and one of its ancestors, *B. rapa*, was constructed using SNP Group III (24,193 SNPs) with the same RAxML parameters and outgroups as above. Additionally, the maximum-likelihood phylogeny among all the *B. napus*, *B. oleracea*, and wild C species was calculated with SNP Group IV (23,387 SNPs) with the same pipeline implemented as for the A sub-genome phylogeny.

### Organellar genome assembling and phylogenetic analyses

Three hundred sixteen accessions among these species were also sequenced using genome survey sequencing (GSS) for 2 × 150-bp paired-end reads (Supplementary Data 1). Raw reads were filtered by Trimmomatic (version 0.36)^48^ and then mapped using Bowtie2 (version 2.2.6)^50^ to chloroplast and mitochondrial reference genomes, both which contain the chloroplast or mitochondrial genomes of the six species of the Triangle of U (Supplementary Table 11). Next, paired-end reads mapped to chloroplast reference genomes were kept and used for de novo assembly of chloroplast genomes for each accession with SPAdes (version 3.11.1)^58^. Scaffolds larger than 300 bp were blasted against the *A. thaliana* chloroplast genes (TAIR, Araport11). The sequences which covered whole *A. thaliana* chloroplast genes (gaps and mismatches in genes were allowed) were defined as homologs of *A. thaliana* genes. Meanwhile, the paired-end reads which mapped to the mitochondrial reference genomes were used for de novo assembly and identification of mitochondrial homologs of *A. thaliana* genes for each accession using the same method.

For chloroplast genes, we discarded accessions which recovered less than 100 homologs of *A. thaliana* genes, resulting in 245 accessions with 92 shared chloroplast genes including 62 genes from single copy regions. For mitochondrial genes, all accessions obtained 45–47 mitochondrial homologs of *A. thaliana* genes, with 42 genes shared by all.

Each of the shared 62 single copy chloroplast genes were aligned with MAFFT (version 7.310)^59^, then cleaned (with parameter: -clean 0.5) and concatenated in Phyutility (version 2.2.6)^60^ for a total alignment length of 29,962 bp. Using the same methods, we obtained a 22,937 bp data matrix for mitochondrial sequences. These two concatenated sequences were used to construct two maximum-likelihood phylogenies through RAxML (version 8.2.8) using the GTR-GAMMA substitution model with 100 bootstrap replicates. Bootstrap support values were then calculated using BOOSTER^57^.

### Genetic diversity and divergence analysis

SNP Group I (372,546 SNPs) were used to estimate the average nucleotide diversity of each genetic cluster using vcftools (version 0.1.12b)^61^ with a window size of 100k bp and a step size of 25k bp. To calculate F_ST_ among genetic clusters, we utilized vcftools with a window size of 100k bp and a step size of 25k bp. Linkage disequilibrium (*r*^*2*^) was calculated by PopLDdecay (version 3.30, https://github.com/BGI-shenzhen/PopLDdecay) with the parameters: -MAF 0.05 -MaxDist 3000 -Het 0.88 -Miss 0.25. All calculations and plots were completed in R (version 3.1.3) and RStudio (version 1.0.136).

### Selection analysis

SNP Group I (372,546 SNPs) was phased by Beagle (Version 4.1) with GT input format^62^. After that, XP-CLR^63^ and π ratio (π_WEAm_/π_other_cluster_) methods were used to detect the regions under selective sweeps from the diversification of WEAm to the other genetic clusters. The following parameters were applied to run XP-CLR: -w1 0.005 100 2000 1 -p1 0.7. The mean XP-CLR score was calculated using 20k bp sliding windows with a 10k bp step size. Adjacent 20k bp windows with the top 20% XP-CLR scores were grouped into a single region, and then merged regions in the top 5% of the region-wise highest XP-CLR scores were identified, as in Zhang et al. (2017)^64^. To improve prediction accuracy, we also calculated the π ratio between WEAm and other genetic clusters with 20k bp sliding windows and a step size of 10k bp. Windows with the highest 50% of π ratio were merged as single regions. Finally, regions identified by both XP-CLR and π ratio were kept as selective sweep regions^64^. On average, 93.36% of the selective sweep regions identified by XP-CLR were also identified by π ratio method (Supplementary Table 12). Finally, figures were made using CIRCOS (version 0.69–4)^65^ and R (version 3.1.3).

### Gene expression analysis

After mapping *B. napus* clean reads to the *B. napus* reference genome (version 4.1) and rapeseed pan-transcriptome using TopHat2 (version 2.1.0) with default parameters, the differentially expressed genes were calculated between WEAm and other genetic clusters using the Cufflinks package (version 2.2.1)^66^. Analyses were run with restrictive conditions of |log_2_(ratio)|>1.0 and *P*-value < 0.05. All accessions in the same genetic cluster were treated as replicates. The detailed steps following the Cufflinks RNA-Seq workflow are: Cufflinks to assemble transcripts for each sample; Cuffmerge to construct the final transcriptome; Cuffquant to quantify the mapped reads; and Cuffdiff to calculate the differentially expressed genes.

### Functional annotations and metabolic network analyses

The annotations of *B. napus* genes to *B. rapa*, *B. oleracea*, and *A. thaliana* are the same as in Chalhoub et al. (2014)^1^. To identify additional genes of functional importance in *B. napus*, we aligned coding regions to *B. rapa*, *B. oleracea*, and *A. thaliana* CDS databases using BLASTN (version 2.4.0) with (-evalue 1E-6)^67^. The gene functions and descriptions were downloaded from www.arabidopsis.org. Overrepresented GO terms associated with unique DEGs in each *B. napus* genetic cluster were identified using SeqEnrich^68^ base on hypergeometric statistical tests, GO terms in biological processes, molecular functions, and cellular components, with *P*-values < 0.001 reported.

We have also mapped the *B. napus* gene differential expression patterns to an *A. thaliana* metabolic network, AraGEM v1.2^69^. The nodes in the network represent biochemical reactions that are catalyzed by enzymes. We calculated a pooled fold-change in expression for each node in the metabolic network: for all the *B. napus* DEGs that are orthologous to *A. thaliana* enzyme-coding genes specific to a node, we took the ratio of the sum FPKM expression in the *B. napus* genetic clusters (S, SK, or R) over the sum expression in WEAm, and then computed log_2_(ratio) as the fold-change. The metabolic networks for three genetic clusters were visualized using Gephi (version 0.9.2)^70^. Nodes with expression fold-change >0 (on average overexpressed) are colored in red; those that are associated with underexpressed genes are colored in blue. Clustering coefficients for each node were also calculated using Gephi.

### Reporting summary

Further information on research design is available in the Nature Research Reporting Summary linked to this article.

## Supplementary information

Supplementary Information

Reporting Summary

Description of Additional Supplementary Files

Supplementary Data 1

Supplementary Data 2

Supplementary Data 3

Supplementary Data 4

Supplementary Data 5

Supplementary Data 6

Source Data

## Data Availability

Data supporting the findings of this work are available within the paper and its Supplementary Information files. A reporting summary for this Article is available as a Supplementary Information file. The sequencing data for the RNA-Seq and GSS are available at NCBI Sequence Read Archive (SRA) database SRP128554 under project PRJNA428769. This study also used 102 previously published *B. rapa* transcriptome data sets [SRP072186] for new analyses^18^. The seven SNP groups and the organellar genes are deposited to figshare [https://figshare.com/s/20afe1aa9cc682304163]. The source data underlying Figs. 1a and 3e as well as Supplementary Figs. 1, 2, 3, 4, 14, 17 and 18 are provided as a Source Data file. All other data or supporting files are available from the ﻿corresponding authors upon request.
