# Supplementary material for: Transcriptome and organellar sequencing highlights the complex origin and diversification of allotetraploid Brassica napus
Source: Nat Commun. 2019 Jun 28;10:2878. doi: 10.1038/s41467-019-10757-1 (PMC6599199; doi:10.1038/s41467-019-10757-1)
Supplement: Supplementary file 1 — Supplementary Information [file 41467_2019_10757_MOESM1_ESM.pdf]

Transcriptome and organellar sequencing highlights the complex origin and diversification of allotetraploid *Brassica napus*

An *et al.*

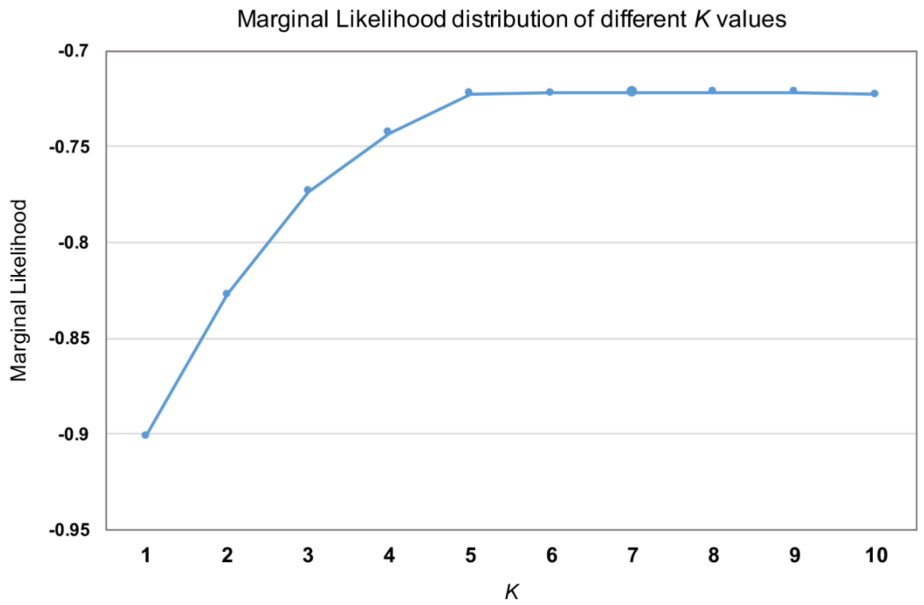

Supplementary Figure 1. Marginal Likelihood value for different *K* values in fastStructure. Based on Darmor-*bzh* reference genome, *K*=7 reaches the maximum marginal likelihood value. Source data are provided as a Source Data file.

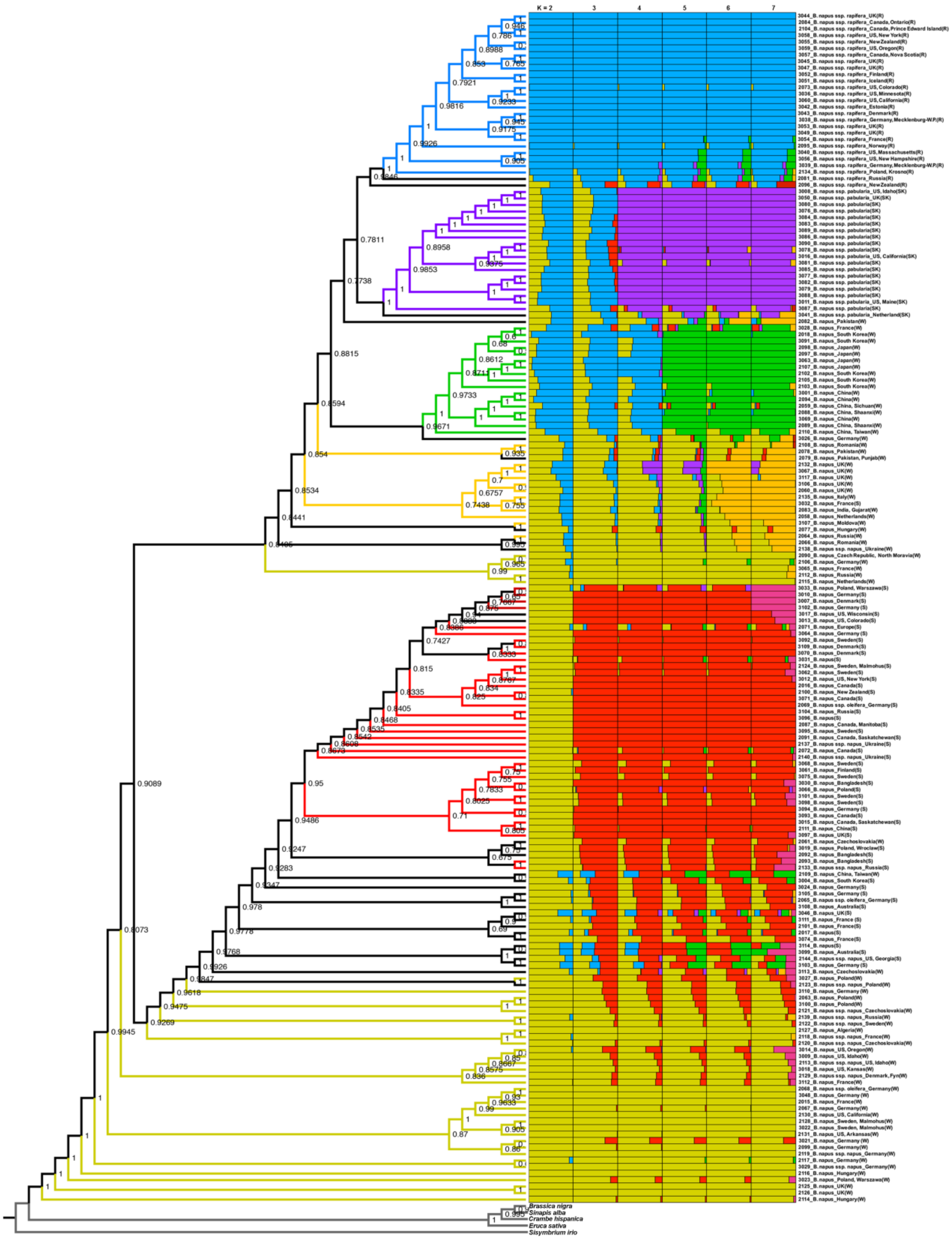

Supplementary Figure 2. Genetic structure and phylogenetic tree of *B. napus*. The phylogenetic tree constructed by maximum-likelihood method. Based on Darmor-*bzh* reference genome. Colors indicates different genetic clusters. Yellow: WEAm (winter rapeseed in Europe and America); Red: S (spring rapeseed); Orange: WEsA (winter rapeseed in Europe and South Asia); Green: WeA (winter rapeseed in East Asia); Purple: SK (Siberian kale); Blue: R(Rutabaga); Dark pink: IntroS (Introgressed spring rapeseed); Black: genetically diverse accessions; Dark grey: outgroups. Source data are provided as a Source Data file.

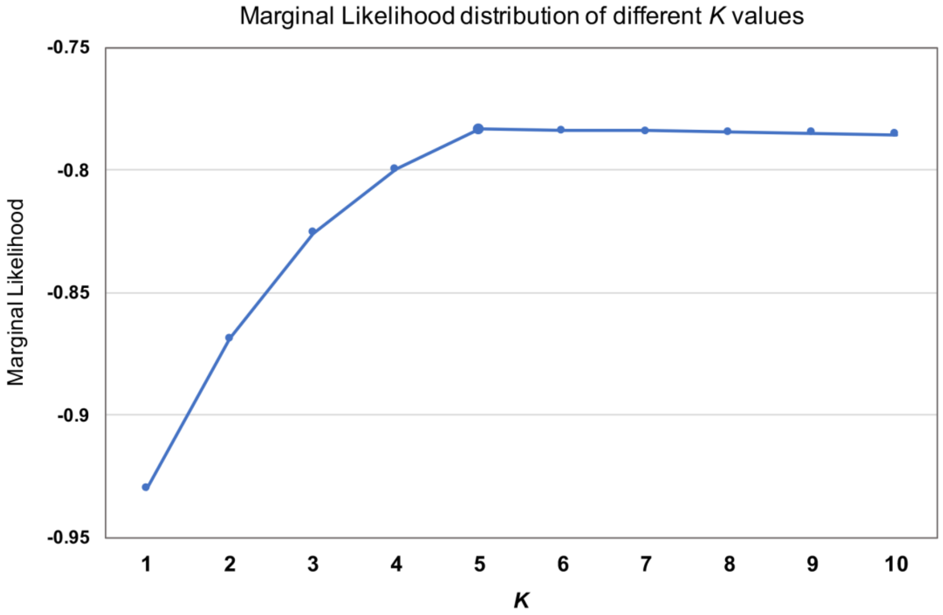

Supplementary Figure 3. Marginal Likelihood value for different *K* values in fastStructure. Based on pan-transcriptome, *K*=5 reaches the maximum marginal likelihood value. Source data are provided as a Source Data file.

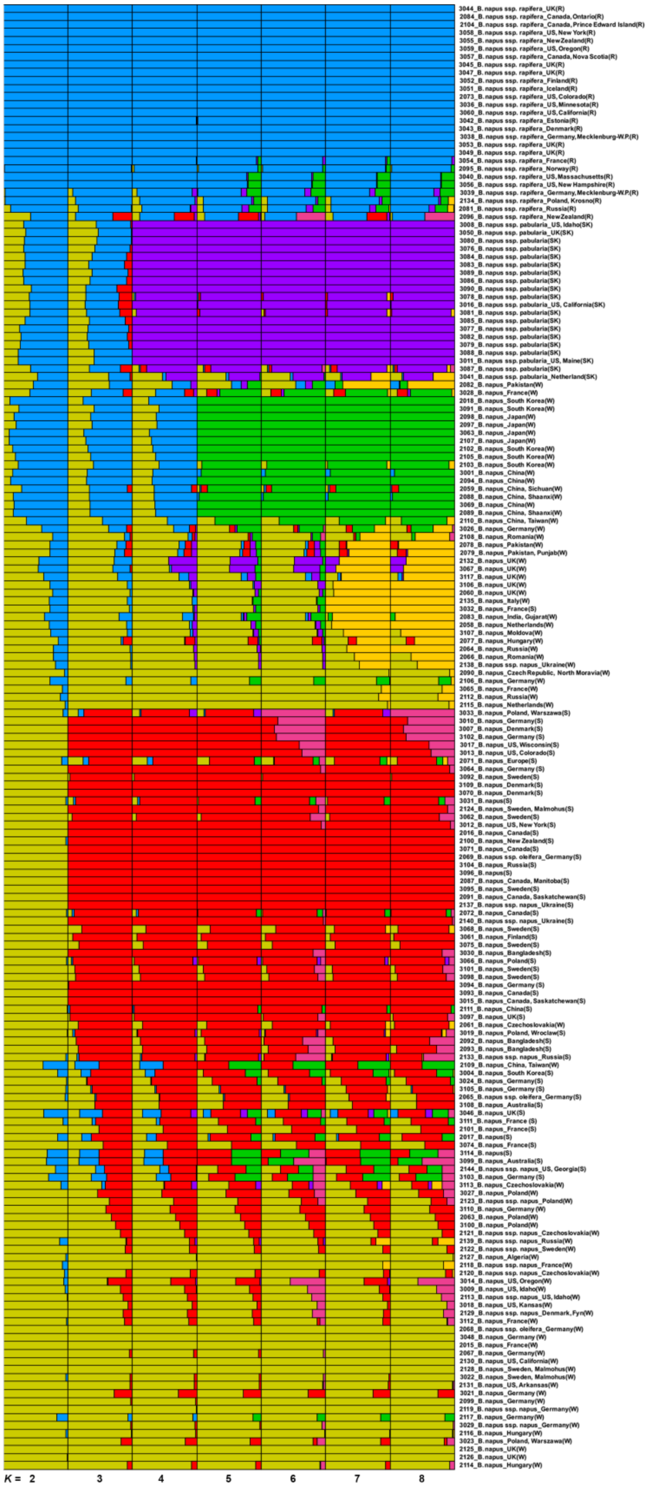

Supplementary Figure 4. Genetic structure of *B. napus* based on pan-transcriptomes. Colors indicates different genetic clusters. Yellow: WEAm (winter rapeseed in Europe and America); Red: S (spring rapeseed); Orange: WEsA (winter rapeseed in Europe and South Asia); Green: WeA (winter rapeseed in East Asia); Purple: SK (Siberian kale); Blue: R(Rutabaga); Dark pink: IntroS (Introgressed spring rapeseed). Source data are provided as a Source Data file.

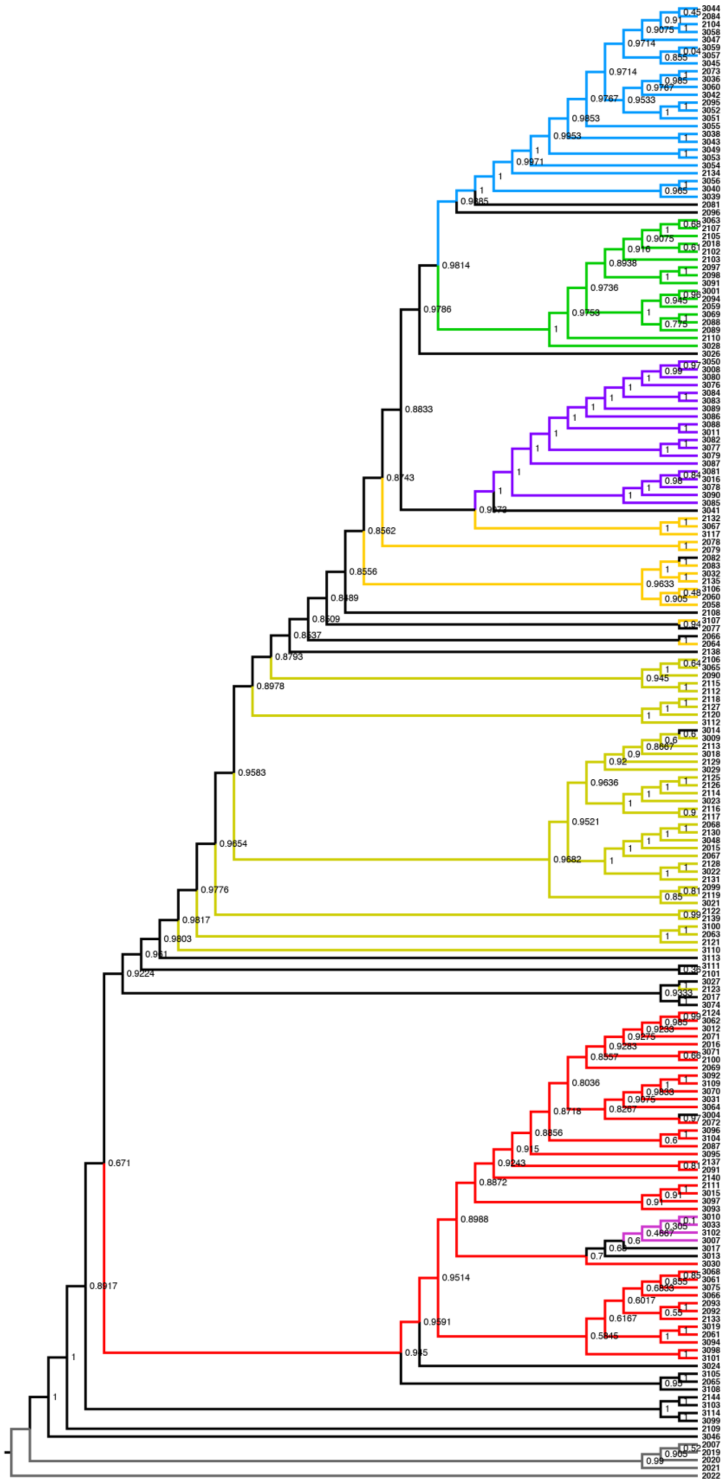

Supplementary Figure 5. Phylogenetic tree of *B. napus* based on pan-transcriptomes. The phylogenetic tree constructed by maximum-likelihood method. Colors indicates different genetic clusters. Yellow: WEAm (winter rapeseed in Europe and America); Red: S (spring rapeseed); Orange: WEsA (winter rapeseed in Europe and South Asia); Green: WeA (winter rapeseed in East Asia); Purple: SK (Siberian kale); Blue: R (Rutabaga); Dark pink: IntroS (Introgressed spring rapeseed); Black: genetically diverse accessions; Dark grey: outgroups.

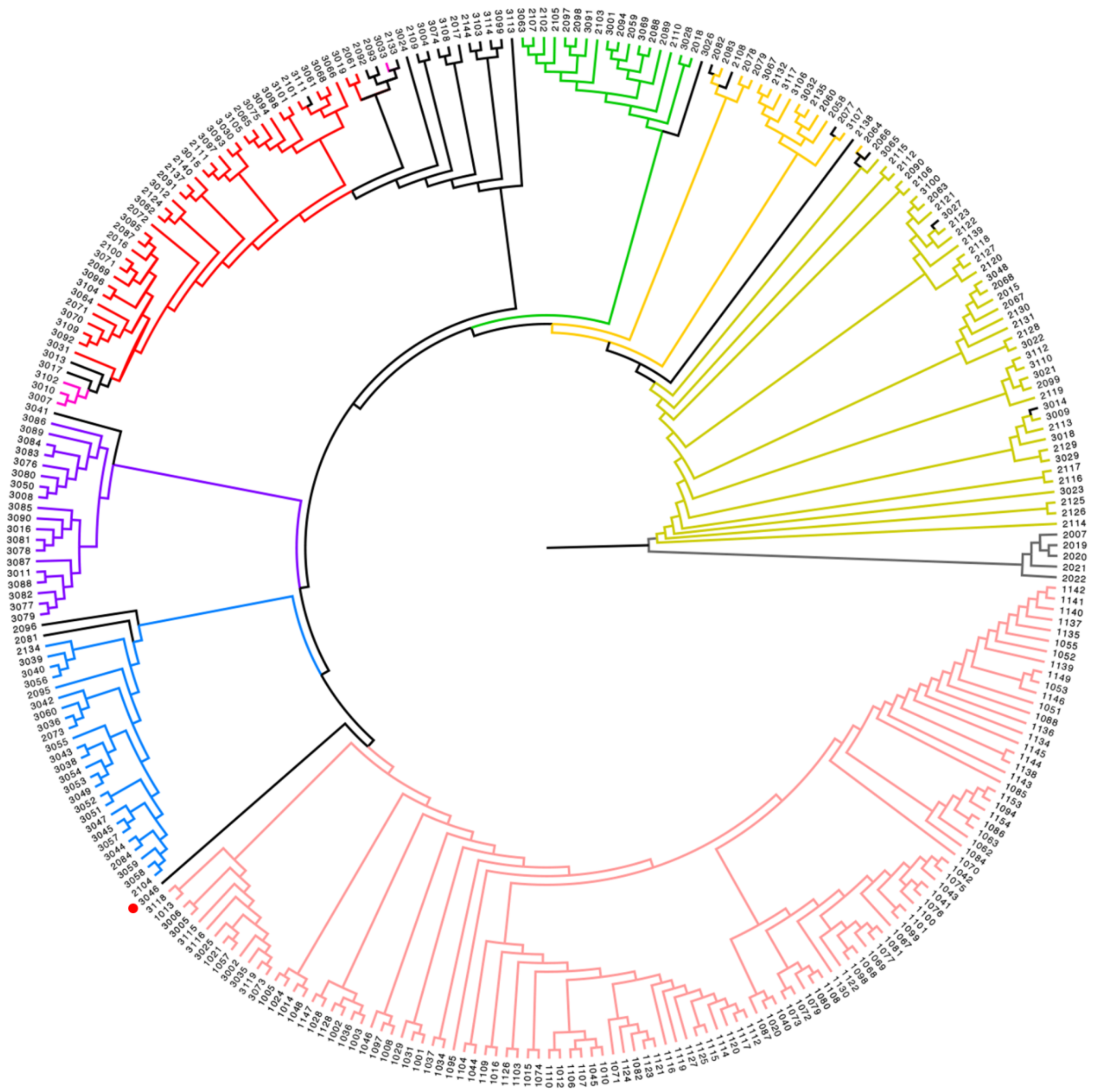

Supplementary Figure 6. Maximum-likelihood phylogenetic tree constructed among *B. napus* and *B. rapa* based on A sub-genome. The red spot indicates a newly resynthesized *B. napus* accession. Colors indicates different genetic clusters. Yellow: WEAm (winter rapeseed in Europe and America); Red: S (spring rapeseed); Orange: WEsA (winter rapeseed in Europe and South Asia); Green: WeA (winter rapeseed in East Asia); Purple: SK (Siberian kale); Blue: R (Rutabaga); Dark pink: IntroS (Introgressed spring rapeseed); Pink: *B. rapa*; Black: genetically diverse accessions; Dark grey: outgroups.

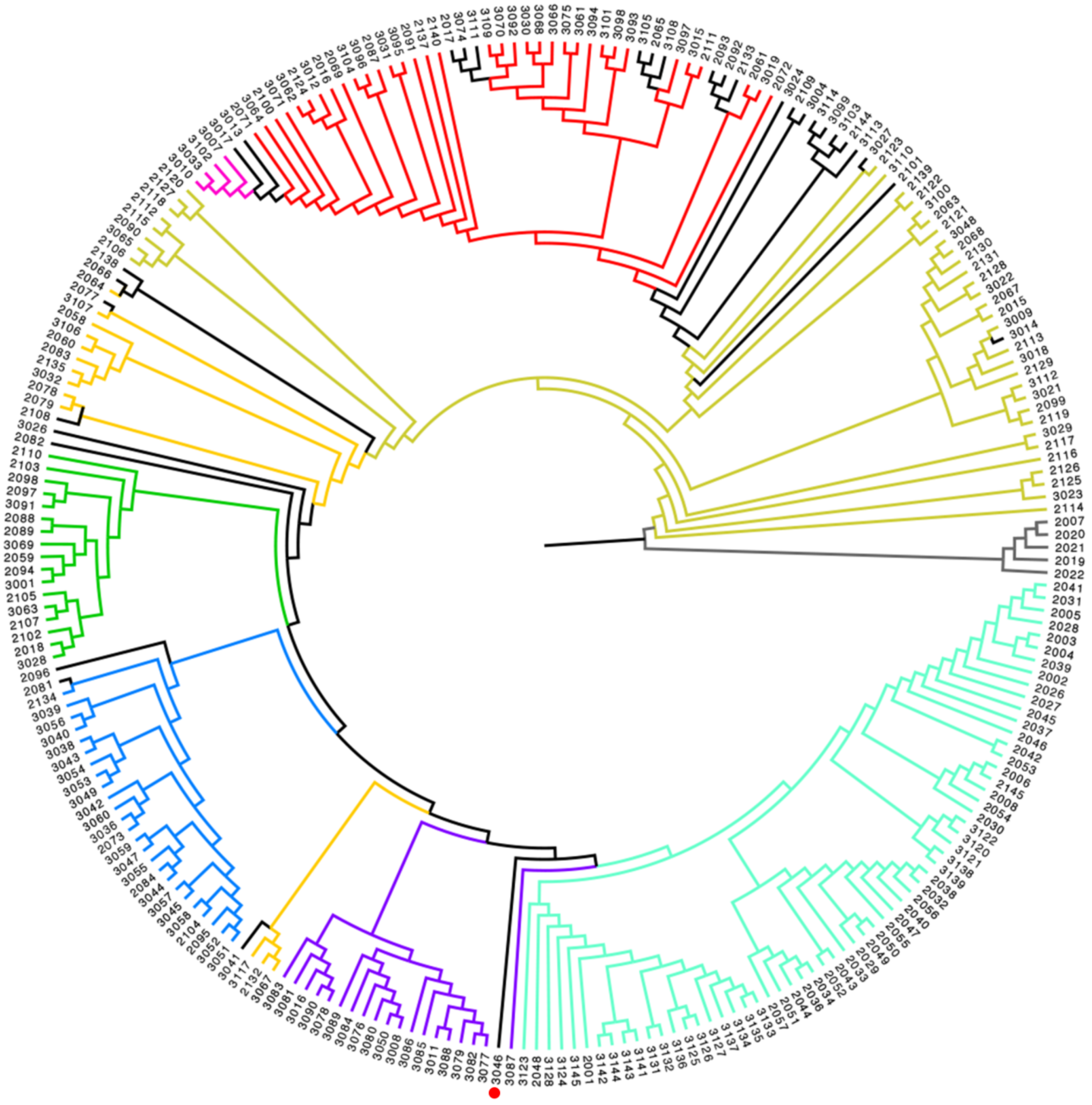

Supplementary Figure 7. Maximum-likelihood phylogenetic tree constructed among *B. napus*, *B. oleracea* and wild C species based on C sub-genome. The Red spot indicates a newly resynthesized *B. napus* accession. Colors indicates different genetic clusters. Yellow: WEAm (winter rapeseed in Europe and America); Red: S (spring rapeseed); Orange: WEsA (winter rapeseed in Europe and South Asia); Green: WeA (winter rapeseed in East Asia); Purple: SK (Siberian kale); Blue: R (Rutabaga); Dark pink: IntroS (Introgressed spring rapeseed); Light blue: *B. oleracea* and wild C species; Black: genetically diverse accessions; Dark grey: outgroups.

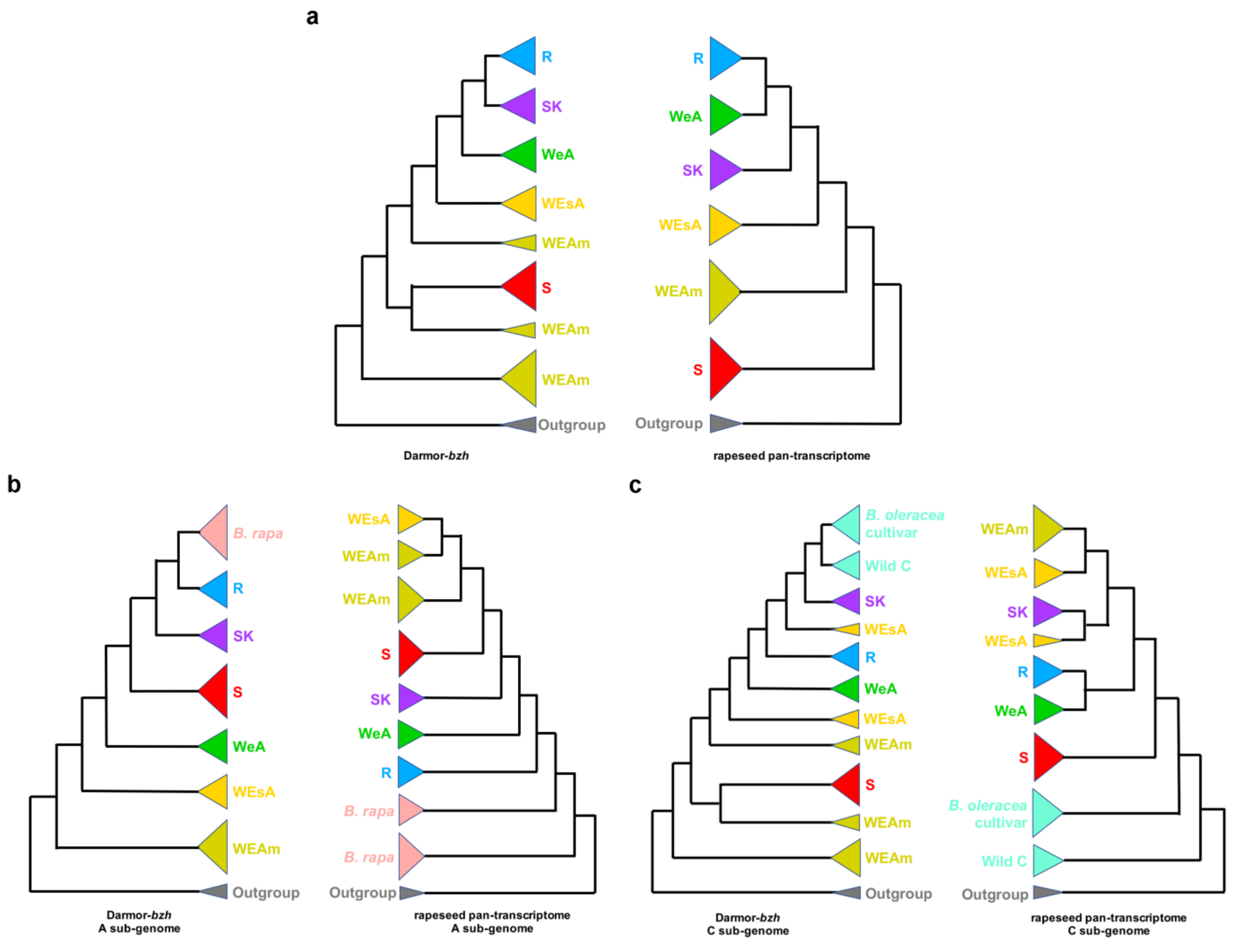

Supplementary Figure 8. Comparison of phylogenetic trees constructed based on Darmor-*bzh* genome and rapeseed pan-transcriptome. a comparison of *B. napus* phylogeny. **b** comparison of A sub-genome phylogeny. **c** comparison of C sub-genome phylogeny. Colors indicates different genetic clusters. Yellow: WEAm (winter rapeseed in Europe and America); Red: S (spring rapeseed); Orange: WEsA (winter rapeseed in Europe and South Asia); Green: WeA (winter rapeseed in East Asia); Purple: SK (Siberian kale); Blue: R (Rutabaga); Pink: *B. rapa*; Light blue: *B. oleracea* and wild C species; Dark grey: outgroups. Note: clades were simplified from full phylogenies for the purpose of comparison.

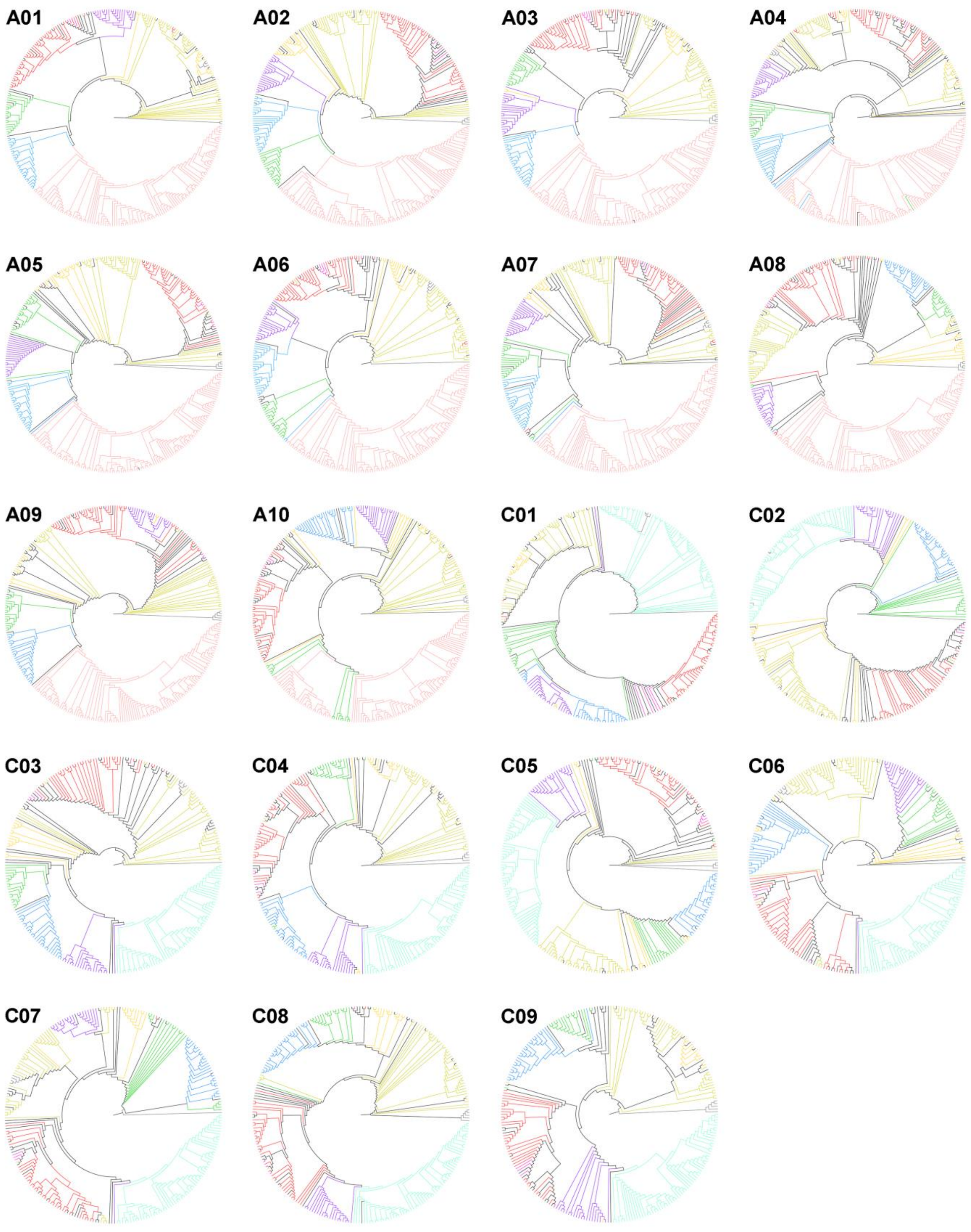

Supplementary Figure 9. Maximum-likelihood phylogenetic tree constructed among *B. napus* and its progenitors based on each chromosome. Colors indicates different genetic clusters. Yellow: WEAm (winter rapeseed in Europe and America); Red: S (spring rapeseed); Orange: WEsA (winter rapeseed in Europe and South Asia); Green: WeA (winter rapeseed in East Asia); Purple: SK (Siberian kale); Blue: R (Rutabaga); Black: genetically diverse accessions; Dark grey: outgroups.

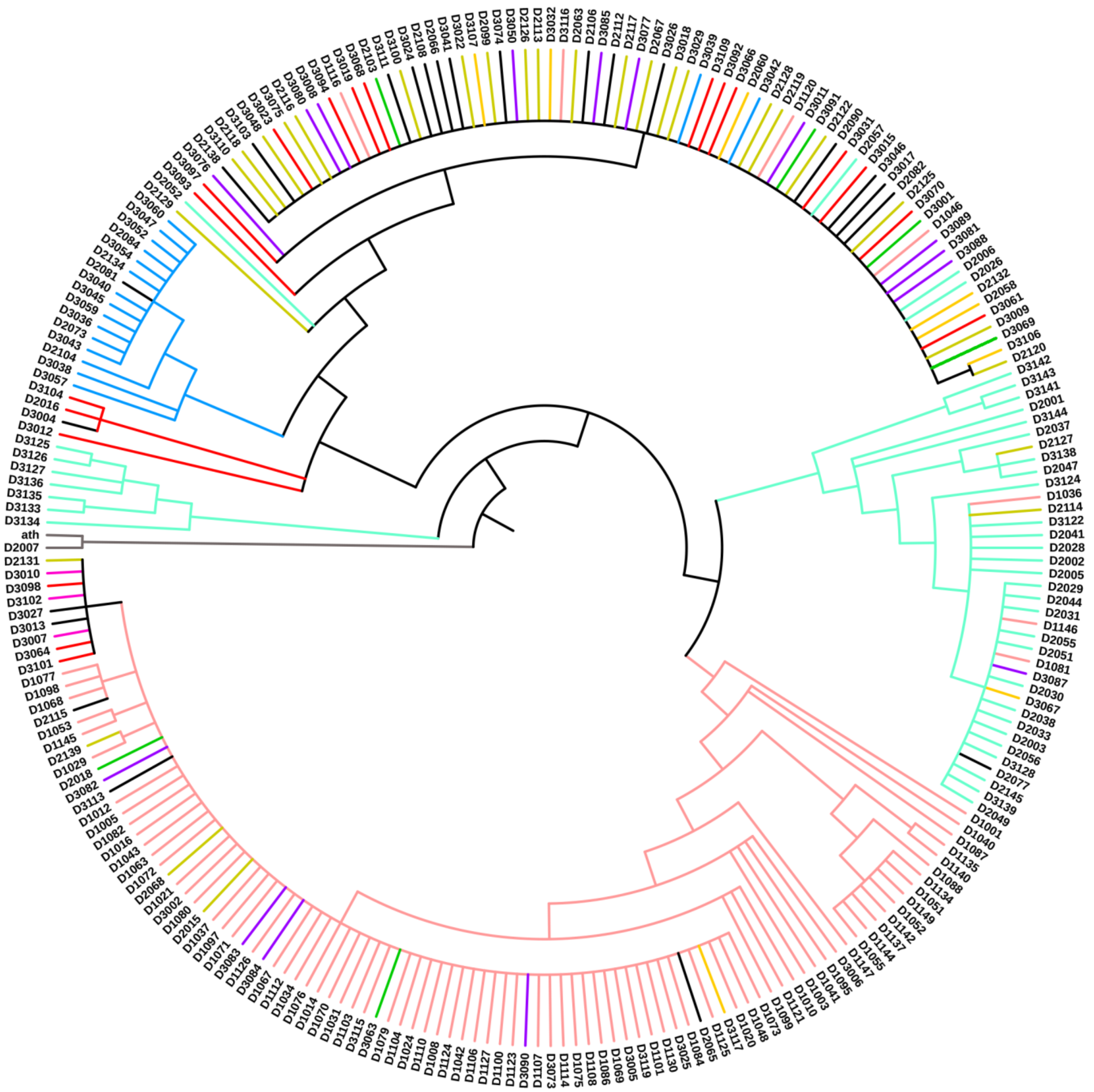

Supplementary Figure 10. Maximum-likelihood phylogenetic tree constructed among *B. napus* and all its progenitor species (*B. rapa*, *B. oleracea* and wild C species) using 62 chloroplast single copy regions homolog genes. ath, *Arabidopsis thaliana.* Colors indicates different genetic clusters. Yellow: WEAm (winter rapeseed in Europe and America); Red: S (spring rapeseed); Orange: WEsA (winter rapeseed in Europe and South Asia); Green: WeA (winter rapeseed in East Asia); Purple: SK (Siberian kale); Blue: R (Rutabaga); Black: genetically diverse accessions; Dark grey, outgroups.

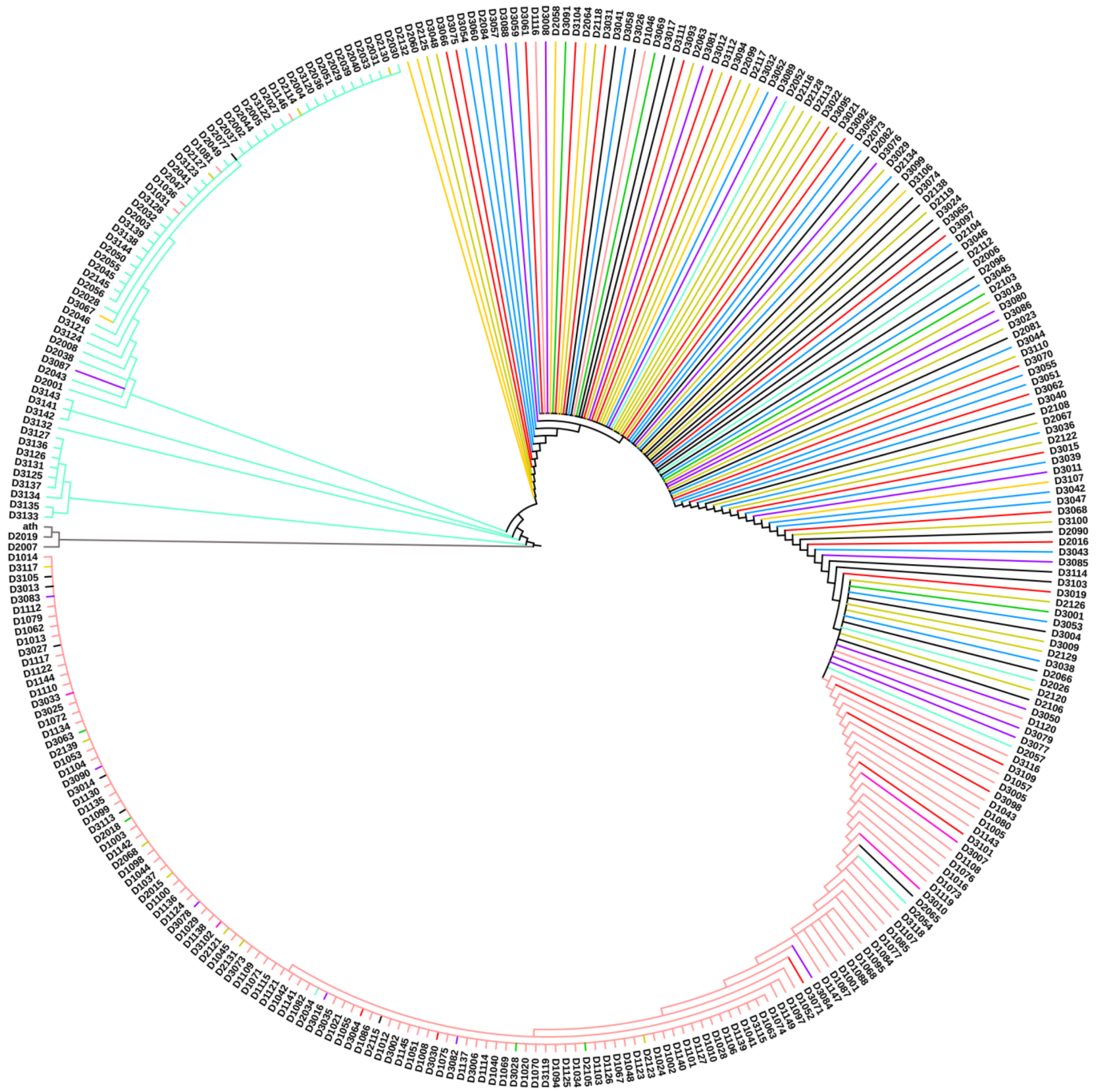

Supplementary Figure 11. Maximum-likelihood phylogenetic tree constructed among *B. napus* and all its progenitor species using 42 mitochondrial homolog genes. ath, *Arabidopsis thaliana*. Colors indicates different genetic clusters. Yellow: WEAm (winter rapeseed in Europe and America); Red: S (spring rapeseed); Orange: WEsA (winter rapeseed in Europe and South Asia); Green: WeA (winter rapeseed in East Asia); Ppurple: SK (Siberian kale); Blue: R (Rutabaga); Black: genetically diverse accessions; Dark grey, outgroups.

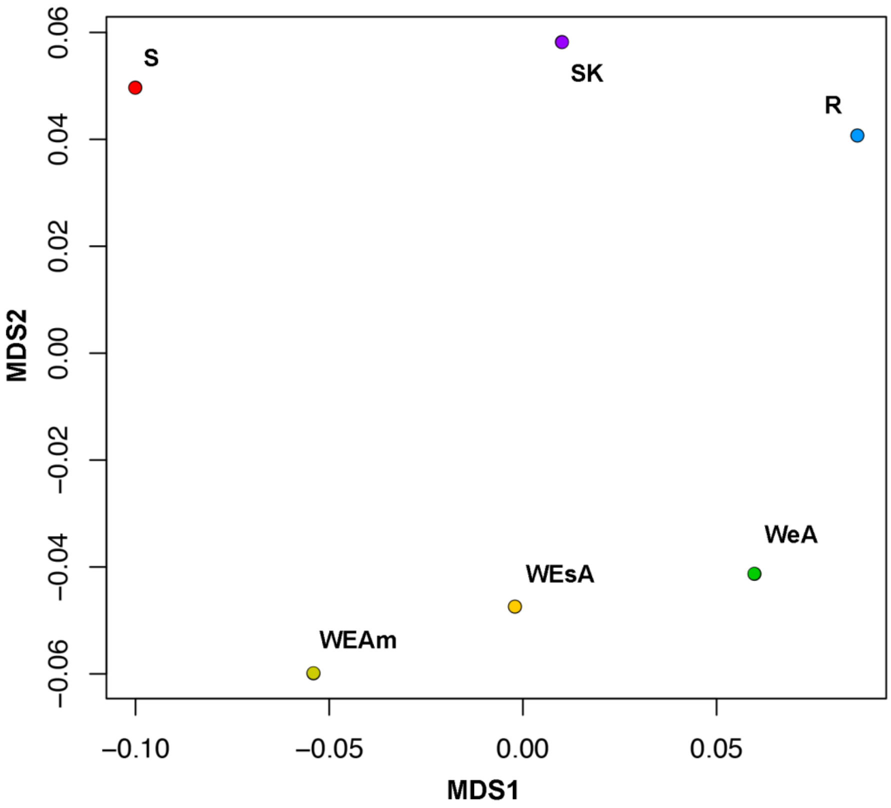

Supplementary Figure 12. Multidimensional scaling (MDS) plot for pairwise F_{ST} matrix among 6 genetic clusters. WEAm: Winter rapeseed in Europe and America; WEsA: Winter rapeseed in Europe and South Asia; S: Spring rapeseed; SK: Siberian kale; R: Rutabaga; WeA: Winter rapeseed in East Asia.

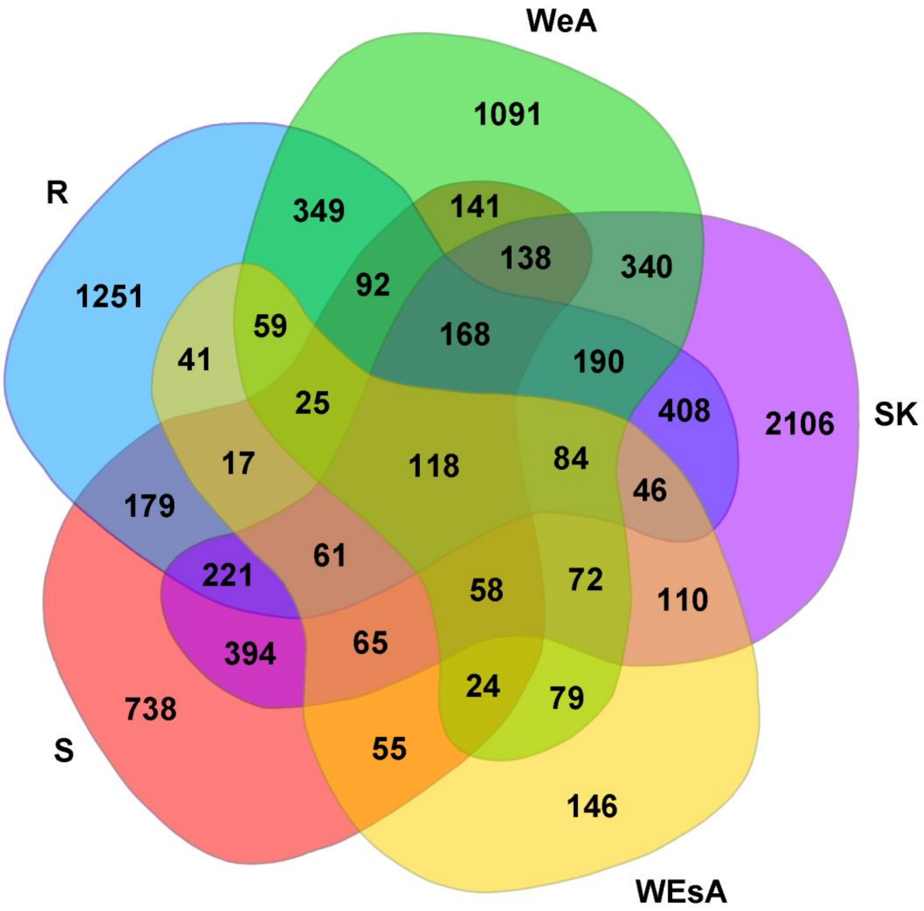

Supplementary Figure 13. The summary of Differential Expressed Genes (DEGs) based on rapeseed pan-transcriptome reference. WEsA: Winter rapeseed in Europe and South Asia; S: Spring rapeseed; SK: Siberian kale; R: Rutabaga; WeA: Winter rapeseed in East Asia.

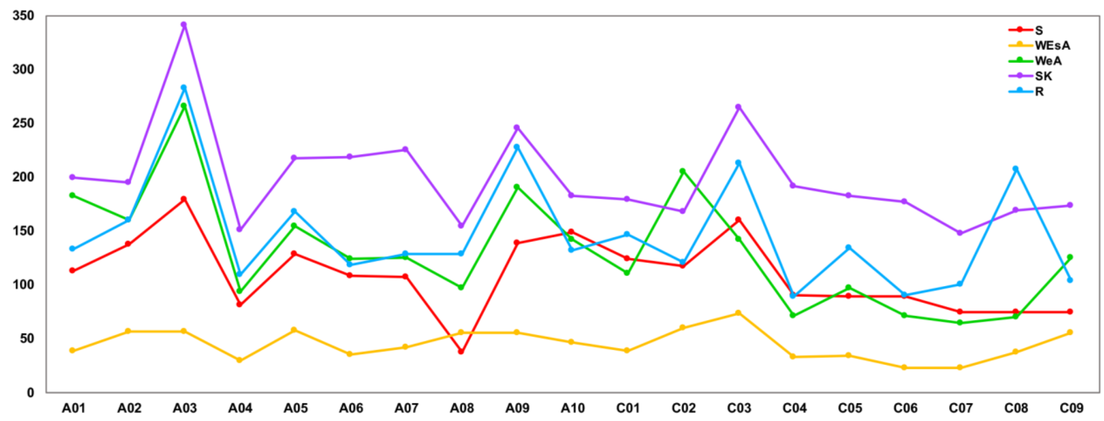

Supplementary Figure 14. Summary of Differentially expressed genes (DEGs) numbers on each chromosome. x axis shows the chromosome name in *B. napus*; y axis shows the DEGs number. S: Spring rapeseed; WEsA: Winter rapeseed in Europe and South Asia; WEAm: winter rapeseed in Europe and America; WeA: Winter rapeseed in East Asia; SK: Siberian kale; R: Rutabaga. Source data are provided as a Source Data file.

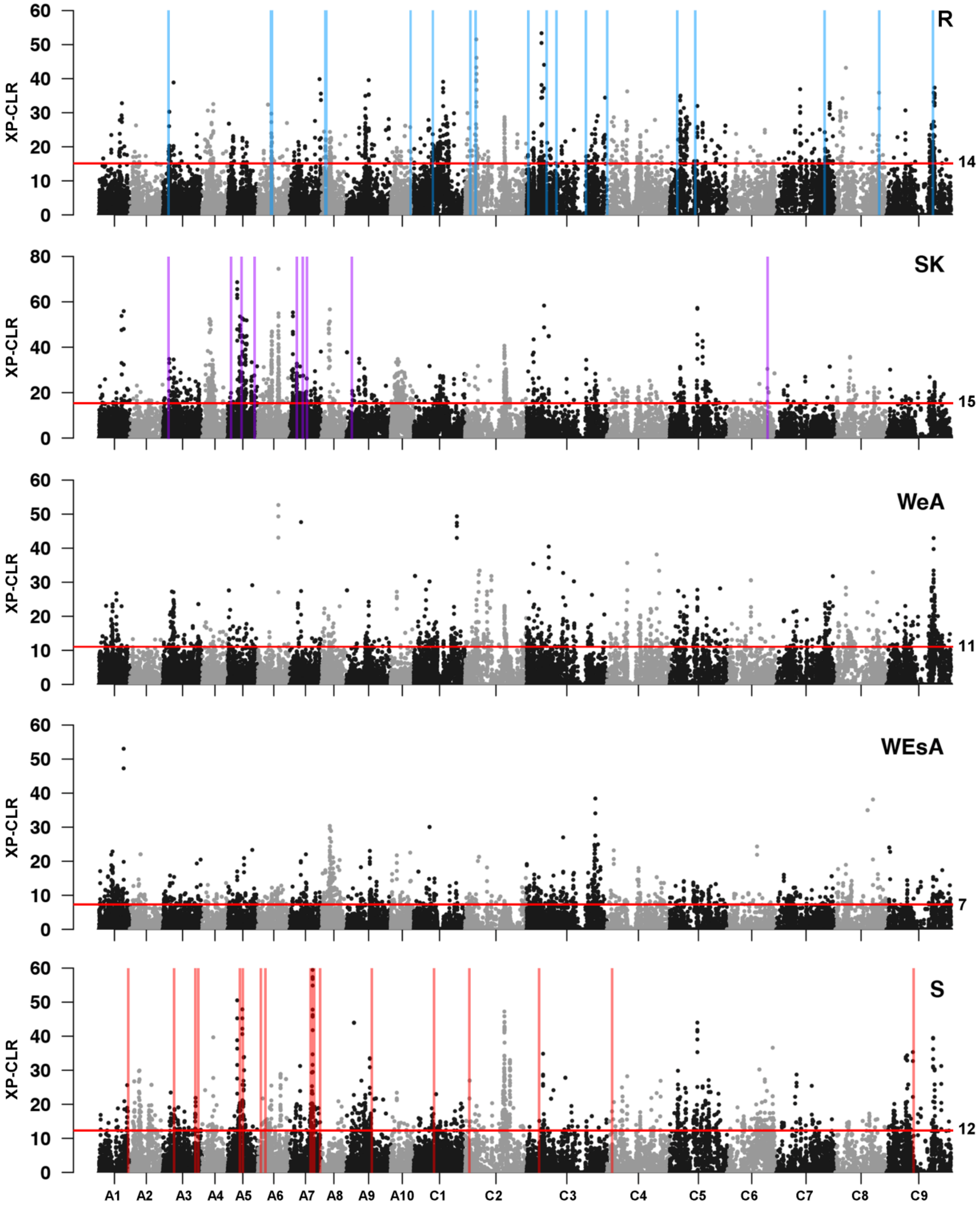

Supplementary Figure 15. Genome wide distributions of selective sweep among 5 genetic clusters based on XP-CLR. Red line: top 5% XP-CLR score; values marked on the right side. R: Rutabaga; SK: Siberian kale; WeA: Winter rapeseed in East Asia; WEsA: Winter rapeseed in Europe and South Asia; S: Spring rapeseed. Colored rectangle showed the selective sweeps with genes and candidate genes functioned in R, SK and S diversification.

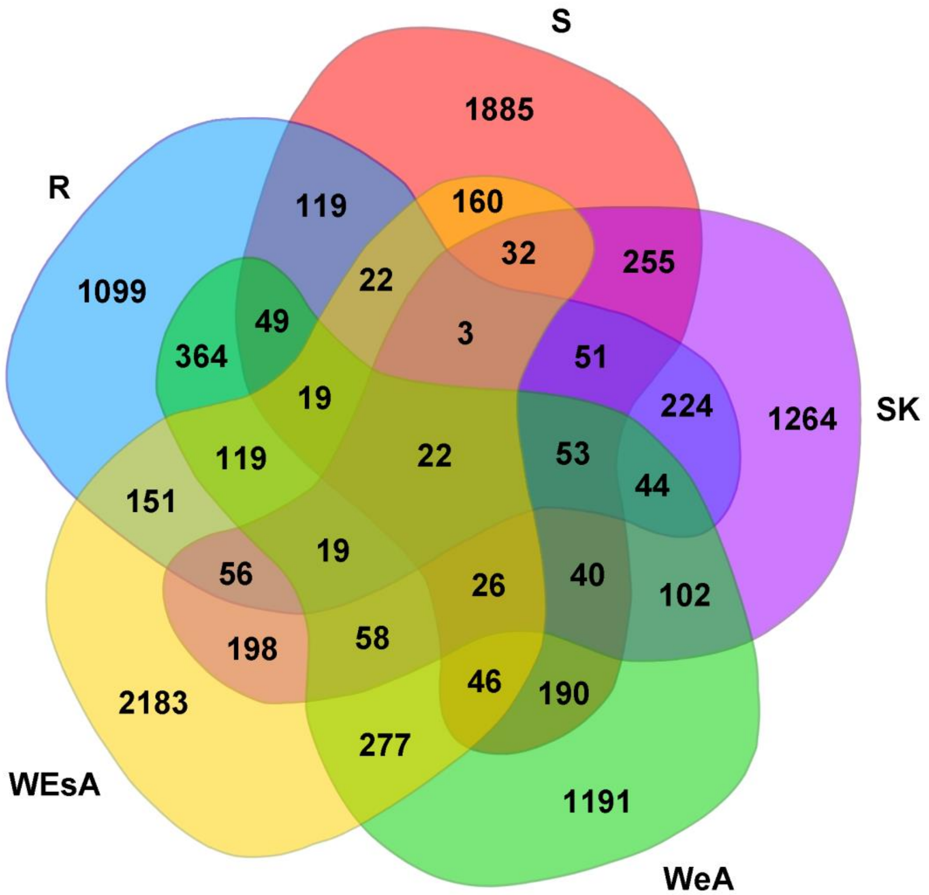

Supplementary Figure 16. The summary of genes under selective sweep among the five genetic clusters. WEsA: Winter rapeseed in Europe and South Asia; S: Spring rapeseed; SK: Siberian kale; R: Rutabaga; WeA: Winter rapeseed in East Asia.

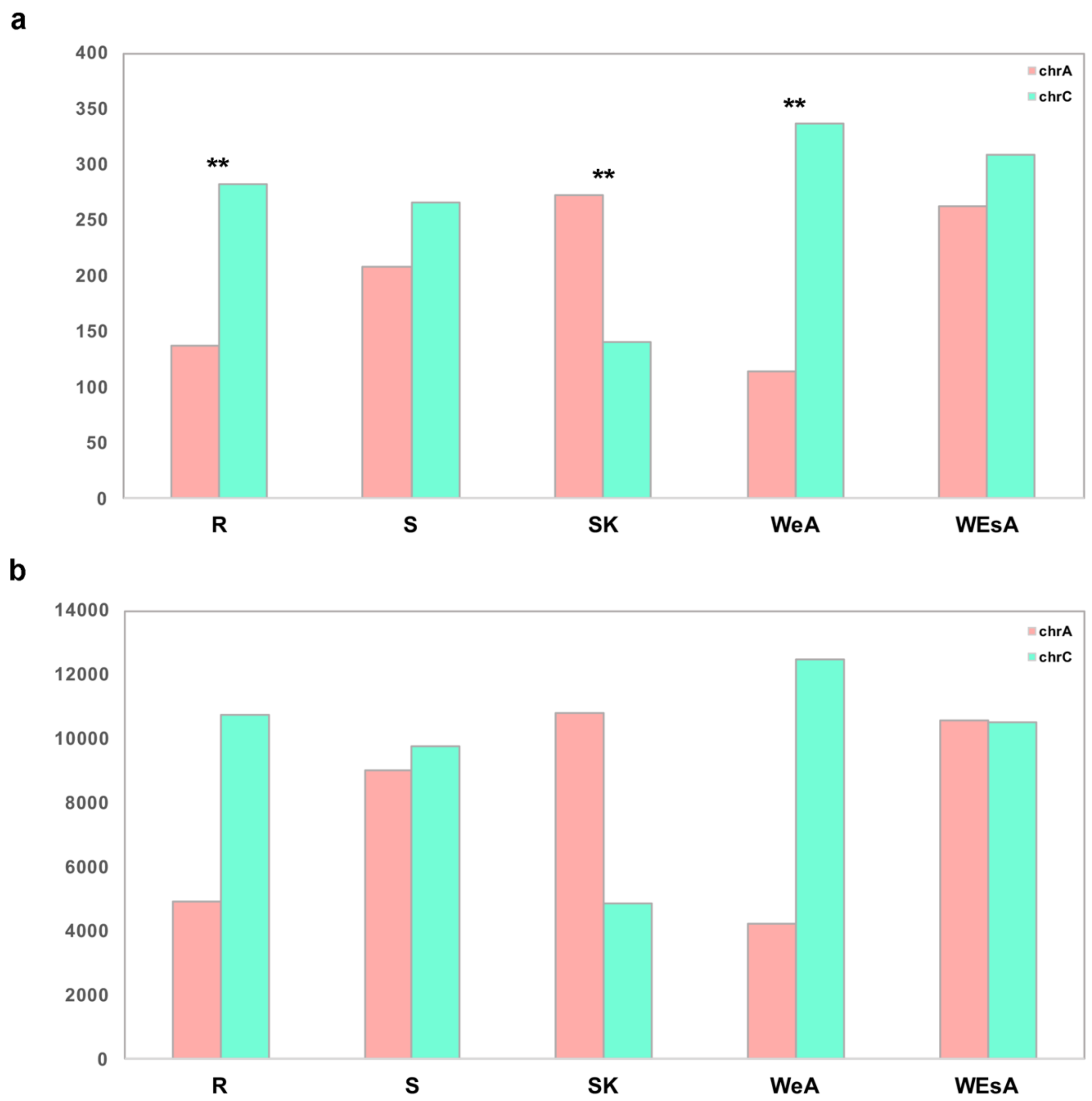

Supplementary Figure 17. The distribution of selective sweep regions in A and C sub-genome. a The number distribution of selective sweep regions. **, p-value < 0.01 (X^2 test). **b** The length distribution of selective sweep regions. The unit for Y-axis is kb. WEsA: Winter rapeseed in Europe and South Asia; S: Spring rapeseed; SK: Siberian kale; R: Rutabaga; WeA: Winter rapeseed in East Asia. Source data are provided as a Source Data file.

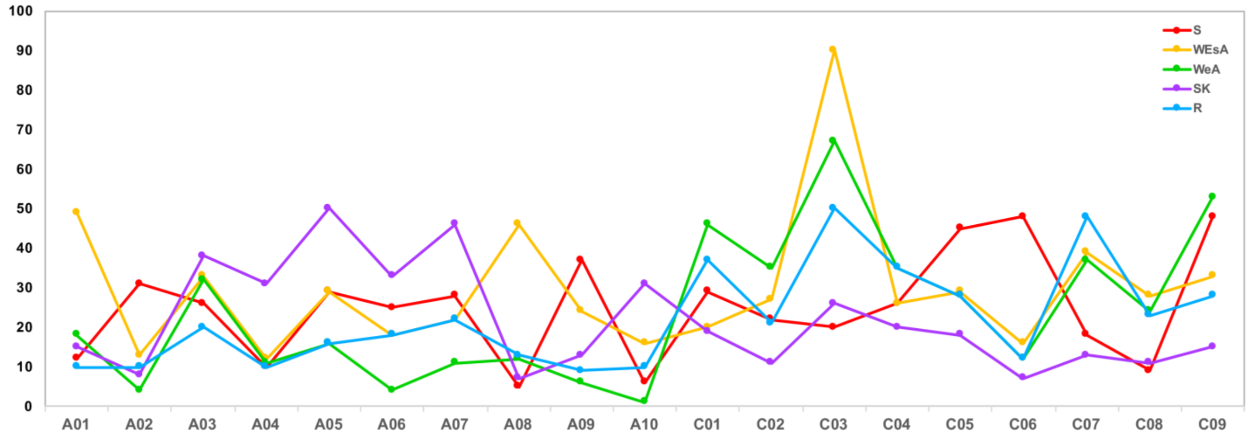

Supplementary Figure 18. Summary of selective sweep regions in each chromosome. Y-axis is the number of selective sweep regions. S: Spring rapeseed; WEsA: Winter rapeseed in Europe and South Asia; WeA: Winter rapeseed in East Asia; SK: Siberian kale; R: Rutabaga. Source data are provided as a Source Data file.

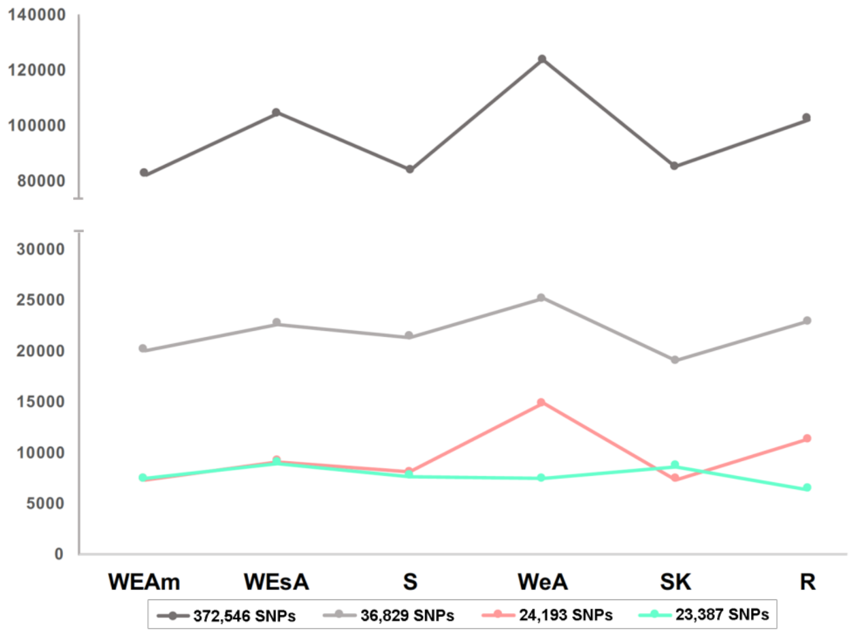

Supplementary Figure 19. Summary of SNPs number in each genetic clusters of the four SNPs dataset used in this study. The order of the x-axis shows the increasing divergence of the genetic clusters compare to WEAm. WEAm: Winter rapeseed in Europe and America; S: Spring rapeseed; WEsA: Winter rapeseed in Europe and South Asia; WeA: Winter rapeseed in East Asia; SK: Siberian kale; R: Rutabaga.

Supplementary Table 1. Single nucleotide polymorphism (SNP) distribution in all *B. napus* accessions.

Chr	SNPs	Density(per kb)	Chr	SNPs	Density(per kb)
chrA01	21,954	0.944	chrC01	15,028	0.387
chrA02	17,419	0.703	chrC02	13,240	0.286
chrA03	32,158	1.080	chrC03	28,317	0.467
chrA04	15,073	0.787	chrC04	16,576	0.339
chrA05	21,987	0.953	chrC05	17,511	0.405
chrA06	25,593	1.049	chrC06	13,559	0.364
chrA07	20,636	0.860	chrC07	15,587	0.348
chrA08	16,113	0.850	chrC08	15,863	0.412
chrA09	29,627	0.875	chrC09	17,701	0.365
chrA10	18,604	1.069			
Total	219,164		Total	153,382	

Supplementary Table 2. Mean pairwise F_{ST} among each genetic cluster

	**WEAm**	**S**	**WEsA**	**SK**	**WeA**	**R**
**WEAm**		0.148	0.077	0.165	0.157	0.183
**S**			0.158	0.178	0.191	0.198
**WEsA**				0.131	0.103	0.150
**SK**					0.163	0.168
**WeA**						0.140
**R**						

WEsA: Winter rapeseed in Europe and South Asia; S: Spring rapeseed; SK: Siberian kale; R: Rutabaga; WeA: Winter rapeseed in East Asia; WEAm: winter rapeseed in Europe and America.

Supplementary Table 3. Summary of differentially expressed genes between WEAm and other genetic clusters

Genetic cluster	Total DEGs	Total up-regulated	Total down-regulated	Unique DEGs	Unique up-regulated	Unique down-regulated
S	2,337	592	1,745	688	188	500
WEsA	956	294	662	151	48	103
WeA	2,773	723	2,050	914	256	658
SK	4,269	1,327	2,942	1,946	732	1,214
R	3,093	754	2,339	1,165	309	856

WEsA: Winter rapeseed in Europe and South Asia; S: Spring rapeseed; SK: Siberian kale; R: Rutabaga; WeA: Winter rapeseed in East Asia; WEAm: winter rapeseed in Europe and America.

Supplementary Table 4. Mean value of nucleotide diversity in different subgroups based on A and C sub-genome

Cluster	A sub-genome	C sub-genome
WEAm	4.944e-05	2.822e-05
WEsA	6.256e-05	3.424e-05
S	5.241e-05	2.635e-05
SK	4.521e-05	3.332e-05
WeA	8.677e-05	2.760e-05
R	7.234e-05	2.271e-05

WEsA: Winter rapeseed in Europe and South Asia, S: Spring rapeseed; SK: Siberian kale; R: Rutabaga; WeA: Winter rapeseed in East Asia; WEAm: winter rapeseed in Europe and America.

Supplementary Table 5. Selected candidate genes for rutabaga diversification.

Chr	Start	End	Gene	Br/Bo Homolog	Ath Homolog	Function	Description
A03	4400001	4510000	*BnaA03g09810D*	*Bra006773*	*AT5G58320*	Kinase interacting (KIP1-like) family protein	expressed in the epidermis of the root meristem and the early expansion zone
A06	10330001	10370000	*BnaA06g18130D*	*Bra018219*	*AT3G46200*	nudix hydrolase homolog 9	modulation of protein N-glycosylation in the roots
A06	10450001	10530000	*BnaA06g18280D*	*Bra038270*	*AT3G45890*	root UVB sensitive-like protein (Protein of unknown function%2C DUF647)	Cheng *et al.* 2016[1]
A08	3300001	3360000	*BnaA08g04000D*	.	*AT1G47240*	NRAMP metal ion transporter 2	root morphogenesis; Mutation impaired root growth
A10	14960001	14990000	*BnaA10g22000D*	*Bra009047*	*AT5G10250*	Phototropic-responsive NPH3 family protein	mutants have defects in primary root growth
C01	13260001	13340000	*BnaC01g19230D*	*Bo1g047910*	*AT4G27060*	ARM repeat superfamily protein	the orientation of cortical microtubules and the direction of organ growth
C02	3260001	3290000	*BnaC02g06130D*	*Bo2g012660*	*AT5G15600*	SPIRAL1-like4	mutant result of defective anisotropic cell expansion
C02	8100001	8130000	*BnaC02g12760D*	*Bo2g037670*	*AT5G55910*	D6 protein kinase	basipetal auxin transport
C03	1070001	1100000	*BnaC03g02240D*	*Bo3g003470*	*AT5G05730*	anthranilate synthase alpha subunit 1	forms a link between ethylene signaling and auxin synthesis in roots
C03	14330001	14360000	*BnaC03g25570D*	*Bo4g196030*	*AT2G46070*	mitogen-activated protein kinase 12	negative regulator of auxin signaling
C03	14330001	14360000	*BnaC03g25580D*	*Bo3g039070*	*AT2G46070*	mitogen-activated protein kinase 12	negative regulator of auxin signaling
C03	14330001	14360000	*BnaC03g25590D*	*Bo3g039070*	*AT2G46070*	mitogen-activated protein kinase 12	negative regulator of auxin signaling

C03	23410001	23460000	*BnaC03g38180D*	*Bo3g065780*	*AT3G13870*	Root hair defective 3 GTP-binding protein (RHD3)	Cheng *et al.* 2016[1]
C03	44900001	44930000	*BnaC03g55810D*	.	*AT3G46200*	nudix hydrolase homolog 9	modulation of protein N-glycosylation in the roots
C03	57580001	57650000	*BnaC03g67970D*	*Bo3g175620*	*AT4G30290*	xyloglucan endotransglucosylase/hydrolase 19	cellular response to auxin stimulusregions
C05	6290001	6340000	*BnaC05g10910D*	*Bo5g019970*	*AT1G14740*	class I heat shock protein%2C putative (DUF1423)	MP-dependent embryonic root meristem initiation
C05	18300001	18330000	*BnaC05g24110D*	*Bo5g048360*	*AT1G31930*	extra-large GTP-binding protein 3	regulation of root morphological and growth responses
C07	36720001	36830000	*BnaC07g33750D*	*Bo7g104490*	*AT4G16515*	root meristem growth factor	root meristem growth factor
C08	32690001	32820000	*BnaC08g34590D*	*Bo8g100610*	*AT2G23140*	RING/U-box superfamily protein with ARM repeat domain-containing protein	root development, root meristem
C09	32620001	32650000	*BnaC09g29880D*	*Bo9g111400*	*AT5G53540*	P-loop containing nucleoside triphosphate hydrolases superfamily protein	promotion of root distal stem cell (DSC) differentiation
C09	35000001	35080000	*BnaC09g32060D*	*Bo9g120690*	*AT5G55540*	tornado 1	radial pattern of tissue differentiation in the root

“.”: No corresponding gene identified.

Supplementary Table 6. Selected candidate genes for Siberian kale diversification.

Chr	Start	End	Gene	Br/Bo Homolog	Ath Homolog	Function	Description
A03	790001	870000	*BnaA03g01760D*	*Bra005867*	*AT5G05780*	RP non-ATPase subunit 8A	leaf adaxial identity
A05	2560001	2590000	*BnaA05g04870D*	*Bra004913*	*AT2G45450*	binding protein	leaf polarity; adaxial/abaxial axis specification
A05	11260001	11320000	*BnaA05g16600D*	*Bra033869*	*AT1G31880*	DZC domain containing protein	Cheng *et al.* 2016[1]
A05	19150001	19190000	*BnaA05g26050D*	*Bra034692*	*AT3G13300*	Transducin/WD40 repeat-like superfamily protein	cellular response to auxin stimulus
A05	19150001	19190000	*BnaA05g26060D*	*Bra034693*	*AT3G13300*	Transducin/WD40 repeat-like superfamily protein	cellular response to auxin stimulus
A07	5200001	5230000	*BnaA07g04790D*	*Bra015131*	*AT3G25520*	ribosomal protein L5	adaxial/abaxial pattern specification
A07	8870001	8930000	*BnaA07g08860D*	*Bra030038*	*AT1G27340*	Galactose oxidase/kelch repeat superfamily protein	leaf morphology; regulation of auxin mediated signaling
A07	8870001	8930000	*BnaA07g08880D*	*Bra030037*	*AT1G27320*	histidine kinase 3	cytokinin-mediated leaf longevity
A07	11550001	11690000	*BnaA07g13040D*	*Bra012034*	*AT2G27100*	C2H2 zinc-finger protein SERRATE (SE)	adaxial/abaxial pattern formation
A09	4400001	4460000	*BnaA09g08940D*	*Bra037283*	*AT2G17040*	NAC domain containing protein 36	leaf morphogenesis; negative regulate cell size
A09	4400001	4460000	*BnaA09g08950D*	*Bra037283*	*AT2G17040*	NAC domain containing protein 36	leaf morphogenesis; negative regulate cell size
C04	6750001	6780000	*BnaC04g09000D*	*Bo4g034840*	*AT3G52770*	binding protein	adaxial/abaxial axis specification; leaf shaping; leaf
C06	30320001	30350000	*BnaC06g29450D*	*Bo6g020110*	*AT1G68310*	MIP18 family protein (DUF59)	leaf adaxial–abaxial polarity formation

Supplementary Table 7. Selected candidate genes for spring rapeseed diversification.

Chr	Start	End	Gene	Br/Bo Homolog	Ath Homolog	Function	Description
A01	21450001	21510000	*BnaA01g31660D*	*Bra034119*	*AT3G10490*	NAC domain containing protein 52	flowering time control
A03	8860001	8900000	*BnaA03g18840D*	*Bra000165*	*AT2G40080*	EARLY FLOWERING-like protein (DUF1313)	early flowering
A03	24340001	24370000	*BnaA03g47480D*	*Bra019158*	*AT4G25530*	FLOWERING WAGENINGEN	flowering control
A03	26620001	26650000	*BnaA03g51290D*	*Bra024023*	*AT4G31120*	SHK1 binding protein 1	vernalization
A05	9110001	9170000	*BnaA05g14670D*	*Bra038101*	*AT1G53160*	squamosa promoter binding protein-like 4	flower development ; vegetative phase change
A05	11740001	11780000	*BnaA05g17020D*	*Bra040834*	*AT3G22590*	PLANT HOMOLOGOUS TO PARAFIBROMIN	transition from vegetative growth to flowering
A06	3080001	3110000	.	.	*PHYA*		Schiessl *et al.* 2014[2]
A06	3470001	3510000	*BnaA06g06380D*	*Bra019923*	*AT1G10570*	Cysteine proteinases superfamily protein	vegetative to reproductive phase transition of meristem
A06	6510001	6570000	*BnaA06g12580D*	.	*AT1G18450*	actin-related protein 4	early flowering; long-day photoperiodism
A07	15060001	15130000	*BnaA07g18530D*	*Bra003390*	*AT3G59790*	MAP kinase 10	mutant has late flowering in long days
A07	15740001	15910000	*BnaA07g19990D*	*Bra003578*	*AT1G80680*	SUPPRESSOR OF AUXIN RESISTANCE 3	mutant has early-flowering phenotype
A07	17200001	17280000	*BnaA07g22720D*	.	*AT1G73310*	serine carboxypeptidase-like 4	Schiessl *et al.* 2017[3]
A07	17840001	17950000	*BnaA07g23960D*	*Bra003965*	*AT1G70170*	matrix metalloproteinase	mutant has late flowering
A07	22930001	22970000	*BnaA07g33460D*	*Bra015678*	*AT1G77300*	histone-lysine N-methyltransferase	early flower in short days; inhibitor of flowering

A07	22930001	22970000	.	.	*EFS*		Schiessl *et al.* 2014[2]
A09	18540001	18570000	*BnaA09g25530D*	*Bra023153*	*AT1G30970*	zinc finger (C2H2 type) family protein	delayed flowering in winter-annual Arabidopsis
A09	18540001	18570000	.	.	*SUF4*		Schiessl *et al.* 2014[2]
C01	14790001	14840000	*BnaC01g21240D*	*Bo1g052990*	*AT4G17060*	FRIGIDA interacting protein 2	a major determinant of natural variation in Arabidopsis flowering time
C02	3100001	3210000	*BnaC02g05910D*	*Bo2g012420*	*AT5G15160*	BANQUO 2	regulation of flowering time
C03	9610001	9640000	*BnaC03g18800D*	*Bo3g027460*	*AT2G33810*	squamosa promoter binding protein-like 3	regulation of flowering and vegetative phase change
C03	9610001	9640000	.	.	*SPL3*		Schiessl *et al.* 2014[2]
C04	3750001	3770000	*BnaC04g05320D*	*Bo4g025620*	*AT2G40080*	EARLY FLOWERING-like protein (DUF1313)	early flowering
C09	18450001	18490000	*BnaC09g21160D*	*Bo9g072870*	*AT2G03070*	mediator subunit 8	regulation of flower development

“.”: No corresponding gene identified.

Supplementary Table 8. The 22 genes under selective sweeps among all genetic clusters.

Gene	Chr	Start	End	Size	Br/Bo Homolog	Ath Homolog	Sources	Function
BnaA03g12070D	A03	5460001	5470000	10000	.	.	Chalhoub *et al.*[4]	.
BnaA03g12080D					*Bra029048*	.	Chalhoub *et al.*[4]	.
BnaA03g12090D					*Bra029049*	*AT5G54060*	Chalhoub *et al.*[4]	UDP-glucose:flavonoid 3-o-glucosyltransferase
BnaC02g12010D	C02	7410001	7420000	10000	*Bo2g032950*	.	Chalhoub *et al.*[4]	.
BnaC02g12020D					*Bo7g014750*	.	Blast	.
BnaC04g17220D	C04	15260001	15280000	20000	*Bo4g076870*	*AT2G26770*	Chalhoub *et al.*[4]	plectin-like protein
BnaC05g16920D	C05	10630001	10660000	30000	*Bo5g034620*	*AT1G21630*	Chalhoub *et al.*[4]	Calcium-binding EF hand family protein
BnaC05g16930D					*Bo5g034630*	*AT1G21640*	Chalhoub *et al.*[4]	NAD kinase 2
BnaC05g16940D					*Bo5g034640*	*AT1G21651*	Chalhoub *et al.*[4]	zinc ion binding protein
BnaC05g16950D					*Bo5g034640*	.	Blast	.
BnaC05g16960D					*Bo5g034640*	.	Blast	.
BnaC05g16970D					*Bo5g034650*	.	Chalhoub *et al.*[4]	.
BnaC05g16980D					*Bo5g034660*	*AT1G21660*	Chalhoub *et al.*[4]	Chaperone DnaJ-domain superfamily protein
BnaC05g25450D	C05	20440001	20470000	30000	*Bo5g071090*	.	Chalhoub *et al.*[4]	.
BnaC05g25460D					*Bo5g071100*	.	Chalhoub *et al.*[4]	.
BnaC05g25470D					*Bo5g071120*	*AT1G47330*	Chalhoub *et al.*[4]	methyltransferase%2C putative (DUF21)
BnaC05g25480D					*Bo5g071130*	.	Chalhoub *et al.*[4]	.
BnaC05g25490D					*Bo5g071150*	.	Chalhoub *et al.*[4]	.
BnaC06g22290D	C06	24320001	24350000	30000	*Bo6g040610*	*AT1G75580*	Chalhoub *et al.*[4]	SAUR-like auxin-responsive protein family
BnaC06g22300D					*Bo6g040600*	*AT1G75550*	Chalhoub *et al.*[4]	glycine-rich protein
BnaC06g22310D					*Bo6g040590*	*AT1G75540*	Chalhoub *et al.*[4]	salt tolerance homolog2
BnaC06g22320D					*Bo2g155140*	.	Blast	.

“.”: No corresponding gene or annotation identified.

Supplementary Table 9. Summary of contaminated and mislabeled accessions.

Sample ID	Accession	Species	Cultivar	Genome size	Level	Reality
2035	PI 204682	*Brassica oleracea*	No. 190	1.07	cross-species	*B. rapa*
2062	Ames 30186	*Brassica napus*	Tun222	1.03	cross-species	*B. rapa*
2074	PI 169075	*Brassica napus*		1.00	cross-species	*B. rapa*
2075	PI 169080	*Brassica napus*		0.95	cross-species	*B. rapa*
2076	PI 169083	*Brassica napus*		0.98	cross-species	*B. rapa*
2080	PI 251614	*Brassica napus*		0.97	cross-species	*B. rapa*
2085	PI 286418	*Brassica napus*		1.09	cross-species	*B. rapa*
2086	PI 357374	*Brassica napus*	Esenska Mesana	1.19	cross-species	*B. oleracea*
2095	PI 443015	*Brassica napus*	Gry	2.28	in-speices	rutabaga
2096	PI 458607	*Brassica napus*	Doon Major Swede	2.76	in-speices	rutabaga
2136	PI 633123	*Brassica napus*	E94197	0.88	cross-species	*B. rapa*
2141	PI 649135	*Brassica napus*	NU 41737	2.15	cross-species	*B. juncea*
2142	PI 649148	*Brassica napus*	G 32327	1.68	cross-species	*B. rapa*
2143	PI 649153	*Brassica napus ssp. napus*	Span	1.73	cross-species	*B. rapa*
3003	PI 502303	*Brassica napus*	AR-115	1.07	cross-species	*B. rapa*
3046	GT 080920	*Brassica napus ssp. rapifera*	New Hakuran	2.42	in-speices	rapeseed
3048	GT 060523	*Brassica napus ssp. rapifera*	Ceres	2.34	in-speices	rapeseed
3072	GT 050561	*Brassica napus*	CGN_07224	1.14	cross-species	*B. rapa*
3129	BRA3053	*Brassica cretica*		1.35	cross-species	*B. oleracea*
3130	BRA3092	*Brassica cretica*		1.34	cross-species	*B. oleracea*
3140	BRA2918	*Brassica incana*		1.46	cross-species	*B. oleracea*

Supplementary Table 10. SNP distributions of each genetic cluster in different SNP Groups.

Genetic cluster	Cluster size	Group I	Group II	Group III	Group IV
		372,546	36,829	24,193	23,387
WEAm	23	82,246	20,081	7,367	7,456
S	34	83,849	21,377	8,114	7,697
WEsA	12	104,299	22,599	9,083	8,975
WeA	17	123,521	25,131	14,849	7,455
SK	19	85,151	19,046	7,332	8,626
R	25	102,043	22,879	11,266	6,390

Supplementary Table 11. NCBI accessions of the chloroplast and mitochondria reference genomes.

Species	Chloroplast	Mitochondria
Brassica napus	NC_016734	AP006444
Brassica oleracea	KR233156	NC_016118
Brassica rapa	NC_015139	NC_016125
Brassica nigra	NC_030450	NC_029182
Brassica juncea	KT581449	NC_016123
Brassica carinata	KX681672	NC_016120

Supplementary Table 12. Summary of the selective sweep regions identified by XP-CLR and π ratio method.

Genetic cluster	XP-CLR	π ratio	Both
R	459	4215	420
WEsA	616	4068	570
WeA	500	4153	452
S	498	4109	474
SK	424	4083	412

WEsA: Winter rapeseed in Europe and South Asia; S: Spring rapeseed; SK: Siberian kale; R: Rutabaga; WeA: Winter rapeseed in East Asia.

Supplementary References

1. Cheng, F. *et al.* Subgenome parallel selection is associated with morphotype diversification and convergent crop domestication in *Brassica rapa* and *Brassica oleracea*. *Nat. Genet.* **48**, 1218–1224 (2016).
2. Schiessl, S., Samans, B., Hüttel, B., Reinhard, R. & Snowdon, R. J. Capturing sequence variation among flowering-time regulatory gene homologs in the allopolyploid crop species *Brassica napus*. *Front. Plant Sci.* **5**, 404 (2014).
3. Schiessl, S., Huettel, B., Kuehn, D., Reinhardt, R. & Snowdon, R. Post-polyploidisation morphotype diversification associates with gene copy number variation. *Sci. Rep.* **7**, 41845 (2017).
4. Chalhoub, B. *et al.* Early allopolyploid evolution in the post-Neolithic *Brassica napus* oilseed genome. *Science* **345**, 950–953 (2014).
