# Supplementary material for: Transcriptome and organellar sequencing highlights the complex origin and diversification of allotetraploid Brassica napus
Source: Nat Commun. 2019 Jun 28;10:2878. doi: 10.1038/s41467-019-10757-1 (PMC6599199; doi:10.1038/s41467-019-10757-1)
Supplement: Supplementary file 2 — Reporting Summary [file 41467_2019_10757_MOESM2_ESM.pdf]

nature research

Corresponding author(s): J. Chris Pires and Bin Yi

Last updated by author(s): 2019/05/24

Reporting Summary

Nature Research wishes to improve the reproducibility of the work that we publish. This form provides structure for consistency and transparency in reporting. For further information on Nature Research policies, see Authors & Referees and the Editorial Policy Checklist.

Statistics

For all statistical analyses, confirm that the following items are present in the figure legend, table legend, main text, or Methods section.

n/a	Confirmed	
☐	☒	The exact sample size (*n*) for each experimental group/condition, given as a discrete number and unit of measurement
☐	☒	A statement on whether measurements were taken from distinct samples or whether the same sample was measured repeatedly
☐	☒	The statistical test(s) used AND whether they are one- or two-sided<br>*Only common tests should be described solely by name; describe more complex techniques in the Methods section.*
☒	☐	A description of all covariates tested
☒	☐	A description of any assumptions or corrections, such as tests of normality and adjustment for multiple comparisons
☒	☐	A full description of the statistical parameters including central tendency (e.g. means) or other basic estimates (e.g. regression coefficient) AND variation (e.g. standard deviation) or associated estimates of uncertainty (e.g. confidence intervals)
☐	☒	For null hypothesis testing, the test statistic (e.g. F, t, r) with confidence intervals, effect sizes, degrees of freedom and P value noted<br>*Give P values as exact values whenever suitable.*
☒	☐	For Bayesian analysis, information on the choice of priors and Markov chain Monte Carlo settings
☒	☐	For hierarchical and complex designs, identification of the appropriate level for tests and full reporting of outcomes
☒	☐	Estimates of effect sizes (e.g. Cohen's *d*, Pearson's *r*), indicating how they were calculated

Our web collection on statistics for biologists contains articles on many of the points above.

Software and code

Policy information about availability of computer code

Data collection	No software was used to collect the data.
Data analysis	All softwares used in this study are open source. The name and version of the softwares we used are mentioned in our manuscript, eg. Tophat, GATK, RAxML, XP-CLR, Circos and Samtools et al.

For manuscripts utilizing custom algorithms or software that are central to the research but not yet described in published literature, software must be made available to editors/reviewers. We strongly encourage code deposition in a community repository (e.g. GitHub). See the Nature Research guidelines for submitting code & software for further information.

Data

Policy information about availability of data

All manuscripts must include a data availability statement. This statement should provide the following information, where applicable:

- Accession codes, unique identifiers, or web links for publicly available datasets
- A list of figures that have associated raw data
- A description of any restrictions on data availability

All RNA-seq and GSS raw data is available at NCBI Sequence Read Archive (SRA) database SRP128554 under project PRJNA428769.

Field-specific reporting

Please select the one below that is the best fit for your research. If you are not sure, read the appropriate sections before making your selection.

☐ Life sciences ☐ Behavioural & social sciences ☒ Ecological, evolutionary & environmental sciences

Ecological, evolutionary & environmental sciences study design

All studies must disclose on these points even when the disclosure is negative.

Study description	We studied the origin and diversification processes of an important economic crop, Brassica napus. We explored the relationship between B. napus and its progenitors and the relationship among B. napus subgroups.
Research sample	They are all plant leaf samples mostly from the collection of USDA, collaborators, and our lab.
Sampling strategy	The second youngest leaf was sampled for RNA-seq when the plant has five true leaves.
Data collection	Transcriptome data were collected from our previous study (SRA database) and newly sequenced in this study. GSS data were all newly sequenced by this study.
Timing and spatial scale	All the sampling work were done between 12 PM to 2 PM, and it keeps about one month due to they have different growth rate.
Data exclusions	Some species that were mis-identified were excluded for further analysis because they are labeled as the wrong species. This was determined by genome size and by phenotypic identification
Reproducibility	All data is available on NCBI Sequence Read Archive (SRA) database SRP128554 under project PRJNA428769 and all methods and parameters are clearly states in the methods.
Randomization	For each accession, we planted out four replicates and planted them randomly in four sections of the growth chamber.
Blinding	Blinding is not relevant in this experiment as there are no trials or treatments.

Did the study involve field work? ☐ Yes ☒ No

Reporting for specific materials, systems and methods

We require information from authors about some types of materials, experimental systems and methods used in many studies. Here, indicate whether each material, system or method listed is relevant to your study. If you are not sure if a list item applies to your research, read the appropriate section before selecting a response.

Materials & experimental systems

n/a	Involved in the study
☒	☐ Antibodies
☒	☐ Eukaryotic cell lines
☒	☐ Palaeontology
☒	☐ Animals and other organisms
☒	☐ Human research participants
☒	☐ Clinical data

Methods

n/a	Involved in the study
☒	☐ ChIP-seq
☒	☐ Flow cytometry
☒	☐ MRI-based neuroimaging
