# Supplementary material for: Transcriptome and organellar sequencing highlights the complex origin and diversification of allotetraploid Brassica napus
Source: Nat Commun. 2019 Jun 28;10:2878. doi: 10.1038/s41467-019-10757-1 (PMC6599199; doi:10.1038/s41467-019-10757-1)
Supplement: Supplementary file 3 — Description of Additional Supplementary Files [file 41467_2019_10757_MOESM3_ESM.docx]

**Description of Additional Supplementary Files**

File Name: Supplementary Data 1
Description: Summary of all the samples in this study

File Name: Supplementary Data 2
Description: Summary of the six genetic clusters of *B. napus*

File Name: Supplementary Data 3
Description: Differentially expressed genes between WEAm and other genetic clusters

File Name: Supplementary Data 4
Description: Selective sweeps detected between WEAm and other genetic clusters

File Name: Supplementary Data 5
Description: GO enrichment analysis of unique DEGs

File Name: Supplementary Data 6
Description: Reactions from the metabolic network with high fold-change in expression
